# Multidimensional analysis of TMEM132A in pan-cancer: unveiling its potential as a biomarker for treatment response prediction

**DOI:** 10.7150/jca.96396

**Published:** 2024-06-11

**Authors:** Mingyue Zhang, Shengli Wang, Meihong He, Zhanpeng Zhang, Jie Wu, Hongyan Sun, Hua Zhang, Hengwen Yang

**Affiliations:** 1 Guangdong Provincial Key Laboratory of Tumor Interventional Diagnosis and Treatment, Zhuhai Institute of Translational Medicine, Zhuhai People's Hospital Affiliated with Jinan University, Jinan University, Zhuhai 519000, China.; 2 State Key Laboratory of Bioactive Molecules and Druggability Assessment, The Biomedical Translational Research Institute, Health Science Center (School of Medicine), Jinan University, Guangzhou, 510632, China.; 3 Key Laboratory of Viral Pathogenesis & Infection Prevention and Control (Jinan University), Ministry of Education, Guangzhou, 510632, China.; 4 Department of Metabolic and Bariatric Surgery, The First Affiliated Hospital of Jinan University, Guangzhou, 510632, China.

**Keywords:** TMEM132A, prognosis, immune infiltration, immunological biomarker, Immunotherapy, Pan-cancer

## Abstract

**Background:** TMEM132A is a transmembrane protein that regulates gastric cancer cell malignancy and overall survival in bladder cancer patients. However, while some studies have investigated the involvement of TMEM132A in specific cancers, further systematic studies are required to elucidate its specific mechanisms of action in different cancer types.

**Methods:** We investigated the pan-cancer role of TMEM132A using several databases. We analyzed TMEM132A expression and its correlation with clinical survival, immune checkpoints, tumor stemness score, prognostic value, immunomodulators, genomic profiles, immunological characteristics, immunotherapy and functional enrichment.

**Results:** First, it was observed that TMEM132A expression levels were higher in the majority of tumors compared to non-tumor tissues. In addition, high TMEM132A expression may have a higher prognostic value in some cancers. Furthermore, TMEM132A was significantly associated with immune checkpoints, immunomodulators, prognosis, immunomodulatory genes, tumor stemness score, cell function status and immune infiltration in most tumors. Further analysis of TMEM132A-related gene enrichment, mutation sites and types, RNA modification and genomic heterogeneity showed that the major mutations of TMEM132A were missense mutations and that TMEM132A plays a very important role in UCEC, LUAD and LIHC. Finally, these results suggest that high TMEM132A expression may be associated with a better response to specific immunotherapies.

**Conclusion**: This comprehensive study uncovers an important function for TMEM132A in different types of cancer. It also has the potential to identify TMEM132A as a potential biomarker for predicting treatment response. This may help us to better understand how TMEM132A plays a role in cancer and provide valuable insights for developing personalised treatments.

## 1. Introduction

There are various methods to treat cancer, which can be applied independently or in conjunction with each other. One of these is undergoing surgery, which can directly remove or decrease the size of a tumor and is typically effective against early-stage cancer. However, it also presents potential hazards and complications and may not be suitable for advanced stages or widely disseminated varieties of cancer 1. The use of high-energy radiation to eliminate or restrain cancer cells is appropriate for different types of cancer, it typically preserves the function of surrounding tissues. Nevertheless, it can lead to side effects linked to radiation therapy, including fatigue, inflammation of the skin, and tissue damage, and may necessitate multiple treatments 2. Chemotherapy is also an option, the administration of drugs to eliminate or hinder the proliferation of cancerous cells is applicable to a plethora of cancer types, even those that have metastasized, although it can lead to adverse reactions such as sickness, retching, alopecia, and immune system suppression. It also impacts healthy, normal cells. A therapy targeting specific areas or cells could be considered 3. The use of drugs targeting specific molecules or pathways in cancer cells can reduce damage to healthy cells. However, this approach is not effective for all cancer types and may cause drug resistance and side effects 4. Immunotherapy can activate or enhance the immune system's attack on cancer cells, leading to long-lasting anti-tumor effects. However, not all patients respond effectively to immunotherapy and it may cause immune-related adverse events 5. With regards to cytokine therapy: By introducing synthetic cytokines to enhance or regulate the immune system's function, one can aid in the fight against cancer. However, this treatment method is often accompanied by more side effects, leading to a more complex treatment process with varying effects on a case-by-case basis. It is crucial to note these potential risks and variable outcomes when considering this approach 6, 7. Ultimately, the choice of cancer treatment should be based on the individual circumstances of the patient. Pan-cancer research can provide a deeper understanding of the common characteristics and mechanisms of cancer, regardless of the specific type of cancer being studied, such studies can help identify disease mechanisms, biomarkers and therapies 8. This can lead to more comprehensive and effective treatments. Comprehensive research can help overcome the challenges of cancer treatment, including drug resistance, side effects and cost, and has the potential to improve patient outcomes and survival 9.

TMEM132A, a multifunctional transmembrane protein, has garnered widespread attention in academia for its critical roles in various physiological and pathological processes such as cell adhesion, signal transduction, cancer development, and neural development. The protein possesses unique structural domains, including immunoglobulin-like domains and leucine-rich repeat domains, which provide a structural basis for its functions in cell adhesion and signal transduction 10, 11. Particularly, TMEM132A has been demonstrated to interact with the key protein Wntless (WLS) in the Wnt signaling pathway, thereby influencing the transport and secretion of Wnt ligands, which is crucial for the regulation of the Wnt signaling pathway 12, 13. Moreover, the role of TMEM132A in maintaining cellular homeostasis and responding to stress may be closely related to the treatment of neurodegenerative diseases 14. In the field of neural development, studies have shown that the loss of TMEM132A leads to neural tube closure defects in mice, highlighting its necessity in normal neural development 13, 15, 16. In cancer biology research, the expression levels of TMEM132A are closely associated with the malignant phenotype of gastric cancer. Studies have demonstrated that downregulation of TMEM132A expression can effectively inhibit the proliferation, migration, and invasion abilities of gastric cancer cells, while its overexpression may promote these malignant behaviors 12. Additionally, upregulation of TMEM132A expression is also associated with non-syndromic hearing loss, panic disorders, and the development of various cancers 10, 17. Given the roles TMEM132A plays in multiple diseases, it may emerge as a novel therapeutic target. For instance, inhibitors targeting TMEM132A may hold significant therapeutic potential for treating cancers associated with abnormalities in the Wnt signaling pathway 12. However, current research on TMEM132A primarily focuses on a few types of cancers, and its roles in other tumors remain to be further explored. In order to comprehensively assess the role of TMEM132A in cancer, we conducted an integrated analysis of data from databases such as TCGA, Cancer Cell Line Encyclopedia (CCLE), Genotype-Tissue Expression Project (GTEx), cBioPortal, and Human Protein Atlas (HPA) etc., exploring the expression patterns of TMEM132A and its correlations with clinical survival, immune checkpoints, tumor stemness scores, prognostic value, immune modulators, genomic profiles, immune characteristics, and functional enrichments. We hope to reveal the broad prospects of TMEM132A as a prognostic biomarker and therapeutic intervention target through a systematic analysis of its roles in various cancer types.

## 2. Materials and Methods

### 2.1 TMEM132A expression analysis

The HPA database (https://www.proteinatlas.org) was used to investigate the mRNA expression levels of TMEM132A in normal human tissues, immunohistochemistry and immunofluorescence in normal or tumor tissues. The expression level of the TMEM132A gene in a variety of cancer tissues was obtained using the TIMER2.0 website (http:// timer.cistrome.org/). TMEM132A expression data from normal and tumor samples were obtained from the TCGA (http://cancergenome.nih.gov) and GTEx (http:// commonfund.nih.gov/GTEx/) database.

### 2.2 Pathological stages analysis

We used the "Stage plot "function in the Gene Expression Profiling Interactive Analysis database (GEPIA2; http://gepia.cancer-pku.cn/) to analyze the correlation between TMEM132A expression and tumor stage. The pan-cancer dataset, TCGA Pan-Cancer (PANCAN, N=10,535, G=60,499), was obtained from the UCSC database (https://xenabrowser.net/). From each sample, the expression data of the TMEM132A gene was extracted, and samples with expression levels of 0 were excluded. Subsequently, a log2(x+0.001) transformation was applied to each expression value. To ensure robustness, cancer types with fewer than 3 samples per type were removed, resulting in expression data for 37 cancer types. Differential expression analysis of genes across different clinical stages within each tumor was performed using R software (version 3.6.4). Pairwise significance analysis of differences was conducted using non-paired Student's t-test, while differential testing of multiple sample groups was carried out using analysis of variance 18.

### 2.3 Prognosis analysis

We used GEPIA2 to obtain the OS (Overall Survival) and DFS (Disease-Free Survival) significance map data and survival plots of TMEM132A across all TCGA tumors. Cutoff-high (50%) and cutoff-low (50%) values were used as the expression thresholds to separate the high and low expression cohorts. The log-rank test was used for hypothesis testing. We obtained the pan-cancer dataset, TCGA Pan-Cancer, from the UCSC database. From each sample, we extracted the expression data of the TMEM132A gene. Samples with zero expression levels were excluded, as well as those with follow-up times shorter than 30 days. Subsequently, we applied a log2 transformation to each expression value, incorporating a pseudo-count of 0.001. Additionally, cancer types with fewer than 10 samples per type were removed from the analysis. This preprocessing step resulted in expression data for 39 cancer types, alongside corresponding overall survival data for the samples. To investigate the prognostic relationship between gene expression and survival in each tumor, we utilized the R package survival (version 3.2-7) to construct a Cox proportional hazards regression model with the coxph function. The significance of prognostic differences was evaluated using the Logrank test 18.

### 2.4 Immune regulatory gene, immune checkpoints, and tumor stemness score analysis

The UCSC database, the MuTect2 software and R software was used to analyze the relationship between gene expression and five immune pathway markers (chemokine (41), receptor (18), MHC (21), Immunoinhibitor (24), Immunostimulator (46)). The expression data of the TMEM132A gene and 60 marker genes of two types of immune checkpoint pathway genes (Inhibitory (24) and Stimulatory (36)) extracted from the pan-cancer dataset downloaded from the UCSC database in each sample were calculated for pearson correlation. TMEM132A genes extracted from the pan-cancer dataset downloaded from the UCSC database and DNAss calculated by methylation profile for each tumor were used for the tumor stemness score.

### 2.5 Immune infiltration analysis

We extracted the expression data of the TMEM132A gene in each sample from the pan-cancer dataset downloaded from the UCSC database, extracted the gene expression profile of each tumor, mapped the expression profile to GeneSymbol, and further used the R software package ESTIMATE. stromal, immune, and ESTIMATE scores were calculated for each patient in each tumor based on gene expression. Finally, we obtained immunoinfiltration scores. Pearson's correlation between gene and immunoinfiltration scores in each tumor was calculated using the corr.test function of the R package psych (version 2.1.6) coefficient, to determine the immunoinfiltration score of significant correlation. The TIMER database was used to evaluate the degree of immune cell infiltration in 32 types of cancer.

### 2.6 TMEM132A-related gene analysis

The STRING (https://cn.string-db.org/) was used to retrieve the available experimentally determined TMEM132A-binding proteins (TMEM132A; homo sapiens). We used the "Similar Gene Detection "module of GEPIA2 to screen the top 100 TMEM132A-related target genes. We then used the TIMER2.0 website to obtain the expression of the top 10 target genes in each tumor and displayed them in the form of heatmaps. The sangerbox3.0 database (http://sangerbox.com/home.html) was used to obtain the targeted and correlated genes KEGG pathway, and GO Biological Processes.

### 2.7 Genetic alteration analysis

The mutational character of TMEM132A in different cancers was analyzed using the cBioPortal tool (http://www.cbioportal.org/). And used cBioPortal for further analysis the type and frequency of TMEM132A gene mutations in all tumors. We used the UCSC database to extract the expression data of the TMEM132A gene and 44 marker genes of three types of RNA modification (m1A (10), m5C (13), m6A (21)) genes in each sample, and conducted log2 (x+0.001) for each expression value. The Pearson correlation between TMEM132A and five immune pathway markers was calculated. Used the MuTect2 software deal with samples of all the TCGA level Simple Nucleotide Variation data set. The tmb of each tumor was calculated by using the TMB function of the R software package map tools, and the TMB and gene expression data of the samples were integrated.

### 2.8 The function and enrichment analysis

The correlation between TMEM132A and 14 cancer functional states was analyzed using single-cell sequence data from the CancerSEA website (http://biocc.hrbmu.edu.cn/CancerSEA/home.jsp). Gene co-expression analysis of TMEM132A was performed using LinkedOmics website (https://www.linkedomics.org/login.php). Pearson's test was used to detect the correlation between TMEM132A and the co-expressed genes.

### 2.9 Immunotherapy analysis

The kmplot (https://kmplot.com/analysis/) was used to analyzed the associations between the immune checkpoint blockade (ICB) treatments and TMEM132A. The TISMO website (http://cis.hku.hk/TISIDB/index.php) was used to analyzed the associations between the ICB treatments or Cytokine treatments and TMEM132A.

## 3. Results

### 3.1 TMEM132A was upregulated in multiple human cancers

**Figure 1:**
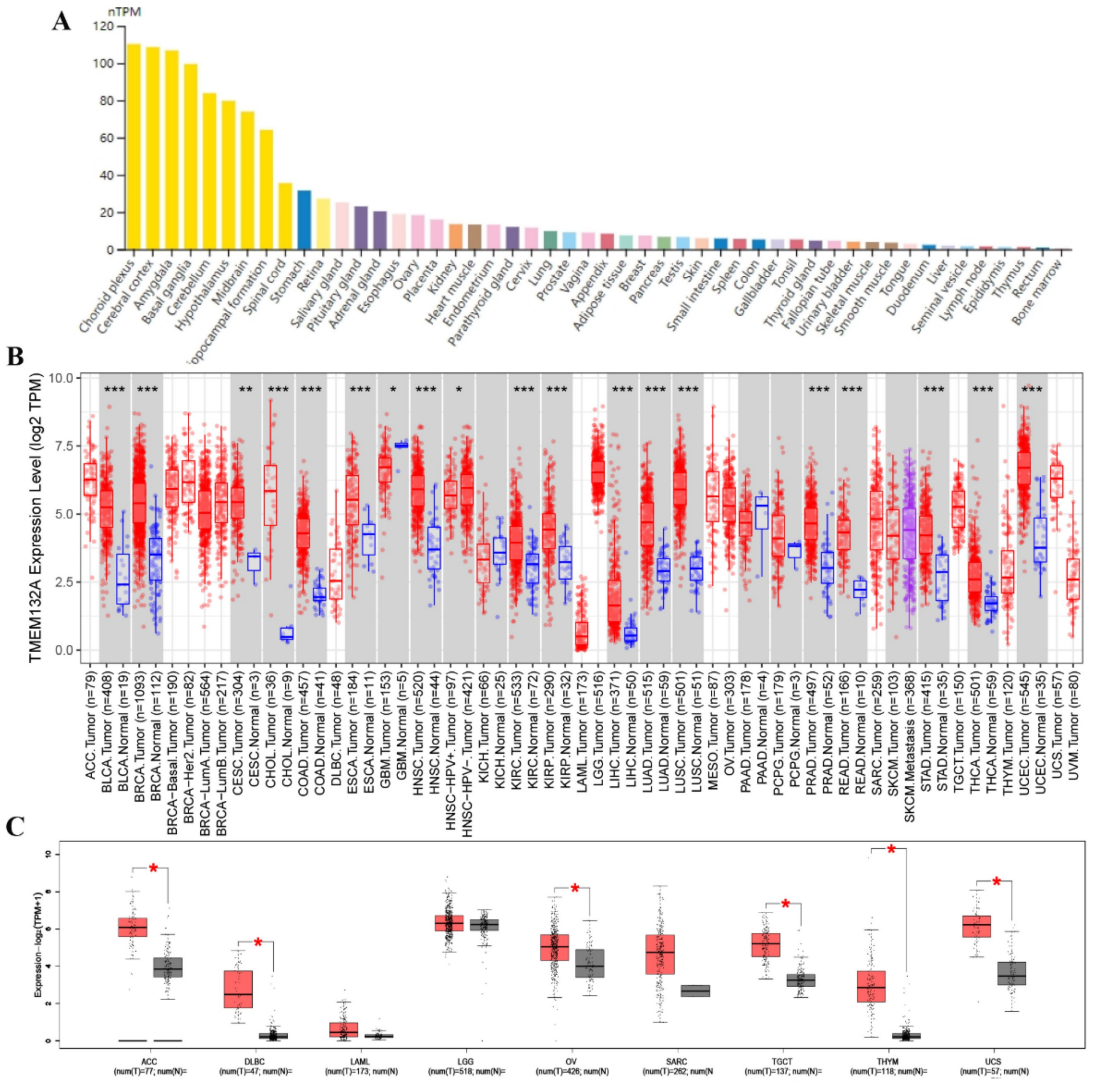
TMEM132A expression in various human normal and tumor tissues. TMEM132A mRNA expression profiles in normal human tissues (A). The expression status of TMEM132A in different tumor types (TCGA database) (*p < 0.05; **p < 0.01; ***p < 0.001) (B). Box plot representation of TMEM132A expression levels comparison in ACC, DLBC, LAML, LGG, OV, SARC, TGCT, THYM, and UCS (TCGA + GTEx database) (*P < 0.05) (C).

We first evaluated mRNA levels in TMEM132A normal human tissue and found its expression in most tissues. Specifically, it exhibited relatively high expression levels in tissues such as the choroid plexus, cerebral cortex, amygdala, basal ganglia, cerebellum, stomach, salivary gland, pituitary gland, ovaries, kidneys, and lungs, etc. (Figure 1A). We further evaluated the expression of TMEM132A in various tumors. We investigated the expression changes of TMEM132A between tumor tissues and matched normal tissues in the TCGA and GTEx database. The mRNA expression of TMEM132A was significantly upregulated in bladder urothelial carcinoma (BLCA), breast invasive carcinoma (BRCA), cervical squamous cell carcinoma (CESC), cholangiocarcinoma (CHOL), colon adenocarcinoma (COAD), esophageal carcinoma (ESCA), Glioblastoma multiforme (GBM), head and neck squamous cell carcinoma (HNSC) kidney renal clear cell carcinoma (KIRC), Kidney renal papillary cell carcinoma (KIRP), Liver hepatocellular carcinoma (LIHC), lung adenocarcinoma (LUAD), lung squamous cell carcinoma (LUSC), prostate adenocarcinoma (PRAD), rectum adenocarcinoma (READ), stomach adenocarcinoma (STAD), thyroid carcinoma (THCA), uterine corpus endometrial carcinoma (UCEC), Adrenocortical carcinoma (ACC), diffuse large B-cell lymphoma (DLBC), ovarian serous cystadenocarcinoma (OV), Testicular Germ Cell Tumors (TGCT), thymoma (THYM) and uterine carcinosarcoma (UCS). However, we did not see significant differences in other tumors, including Acute myeloid leukemia (LAML), brain lower-grade glioma (LGG), and Sarcoma (SARC) (Figure 1B, Figure 1C).

### 3.2 High TMEM132A expression in some cancers may have a higher prognostic value

**Figure 2:**
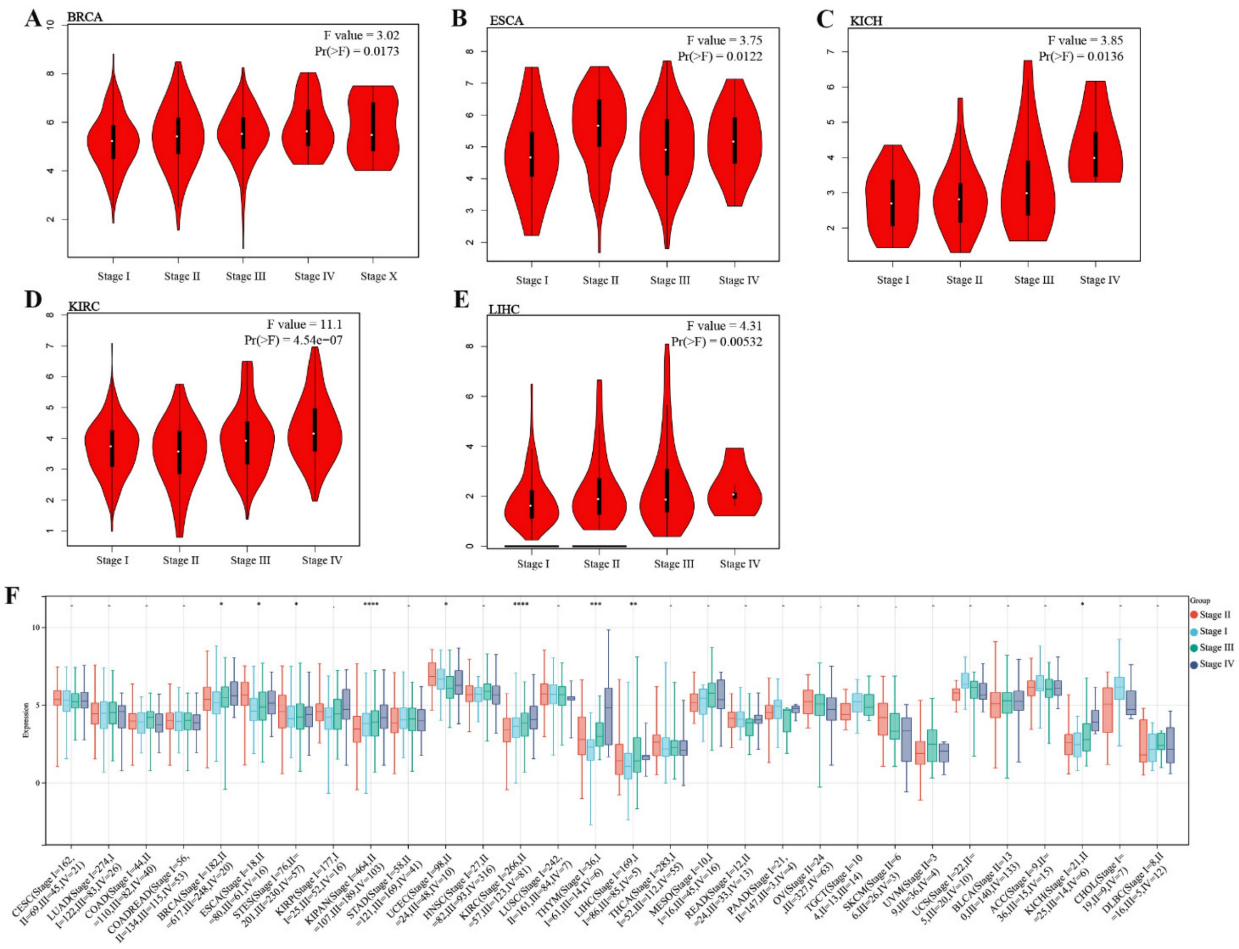
Correlation analysis of TMEM132A and pathological stages. Correlation between TMEM132A expression and pathological stages of BRCA, ESCA, KICH, KIRC and LIHC from TCGA datasets. Log2 (TPM + 1) was applied for log-scale (A-E). The correlation of TMEM132A and pathological stages across all TCGA cancers (F).

We investigated the expression of TMEM132A in different cancer pathological stages and found that in specific tumor types, such as BRCA, ESCA, KICH, KIRC, and LIHC, the expression of TMEM132A was significantly positively correlated with clinical pathological staging (Figure 2A-E). We further analyzed the expression of TMEM132A in different tumor pathological stages using the UCSE database. The results indicated that in specific tumor types (BRCA, ESCA, STES, KIPAN, PRAD, KIRC, LIHC, and BLCA), the expression of TMEM132A was significantly positively correlated with clinical pathological staging (Figure 2F). These results suggest that the expression level of TMEM132A may play a crucial role in the development and progression of certain cancers. Its positive correlation with pathological staging implies its potential as a biomarker for cancer progression, providing new research directions for targeted therapy.

**Figure 3:**
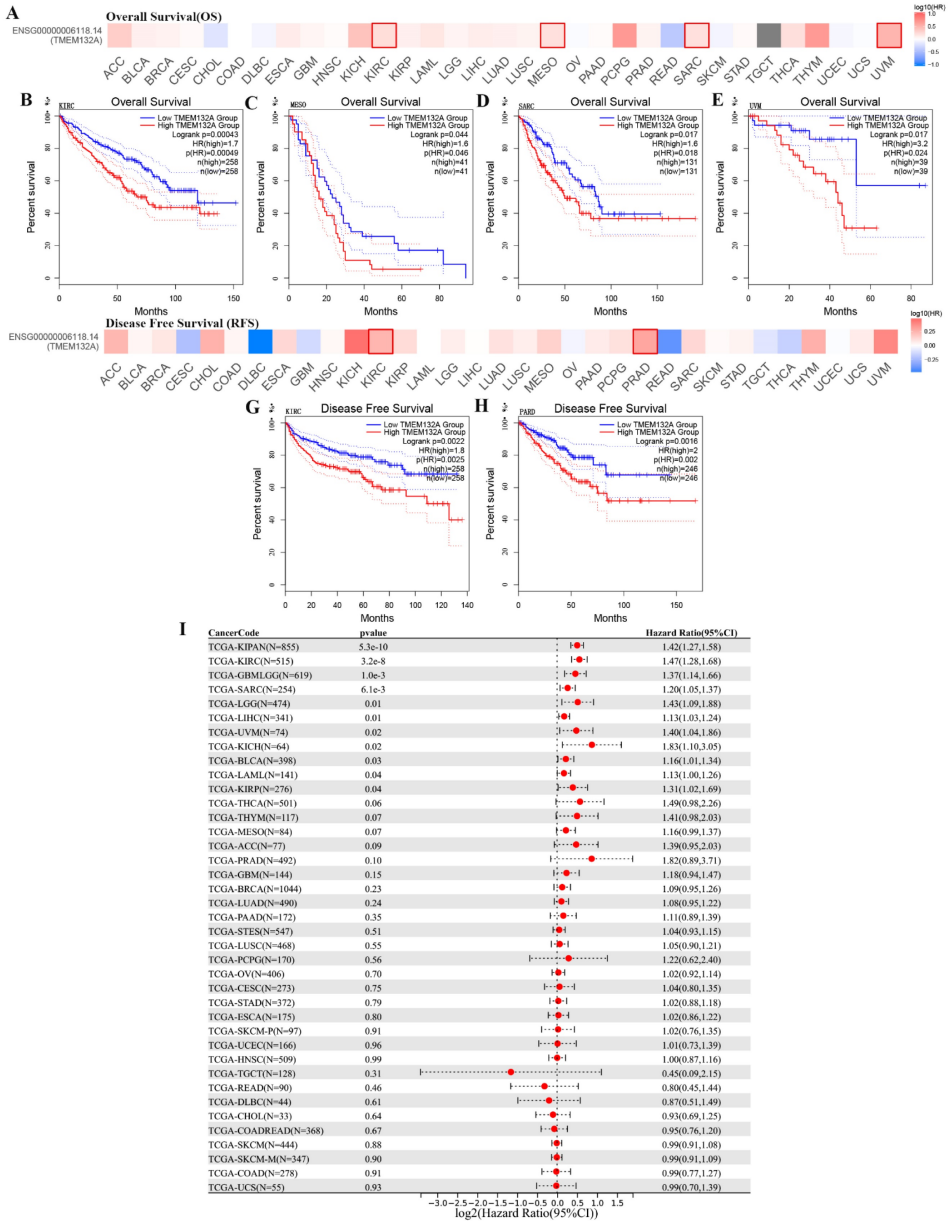
Relationship between TMEM132A expression level and prognosis in TCGA tumors. Relationship between TMEM132A gene expression and overall survival (A), the positive results of the survival map and Kaplan-Meier curves are shown (B-E). Relationship between TMEM132A gene expression and disease-free survival (F) were assessed in all TCGA tumors using EBNAABP2 (G-H). The correlation of TMEM132A and known prognosis across all TCGA cancers (I).

Additionally, comparison of high and low TMEM132A expression cohorts revealed that elevated TMEM132A expression was associated with decreased overall survival (OS) in various tumor types, including KIRC, MESO, SARC, and UVM (Figures 3A-E). Similarly, comparison of high and low TMEM132A expression cohorts showed that increased TMEM132A expression was correlated with decreased disease-free survival (DFS) in KIRC and PRAD (Figures 3F-H). And to use the UCSE database, we observed that high expression in 15 tumor types had a poor prognosis (GBMLGG, LGG, LAML, SARC, KIRP, KIPAN, KIRC, LIHC, UVM, BLCA, KICH) (Figure 3I). The data indicate that elevated TEME132A expression is associated with poor prognosis, suggesting that patients with high TEME132A expression may have higher recurrence rates or poorer survival. This suggests that TEME132A could be used as a potential biomarker for prognosis.

### 3.3 Association between TMEM132A expression and immunomodulatory genes

**Figure 4:**
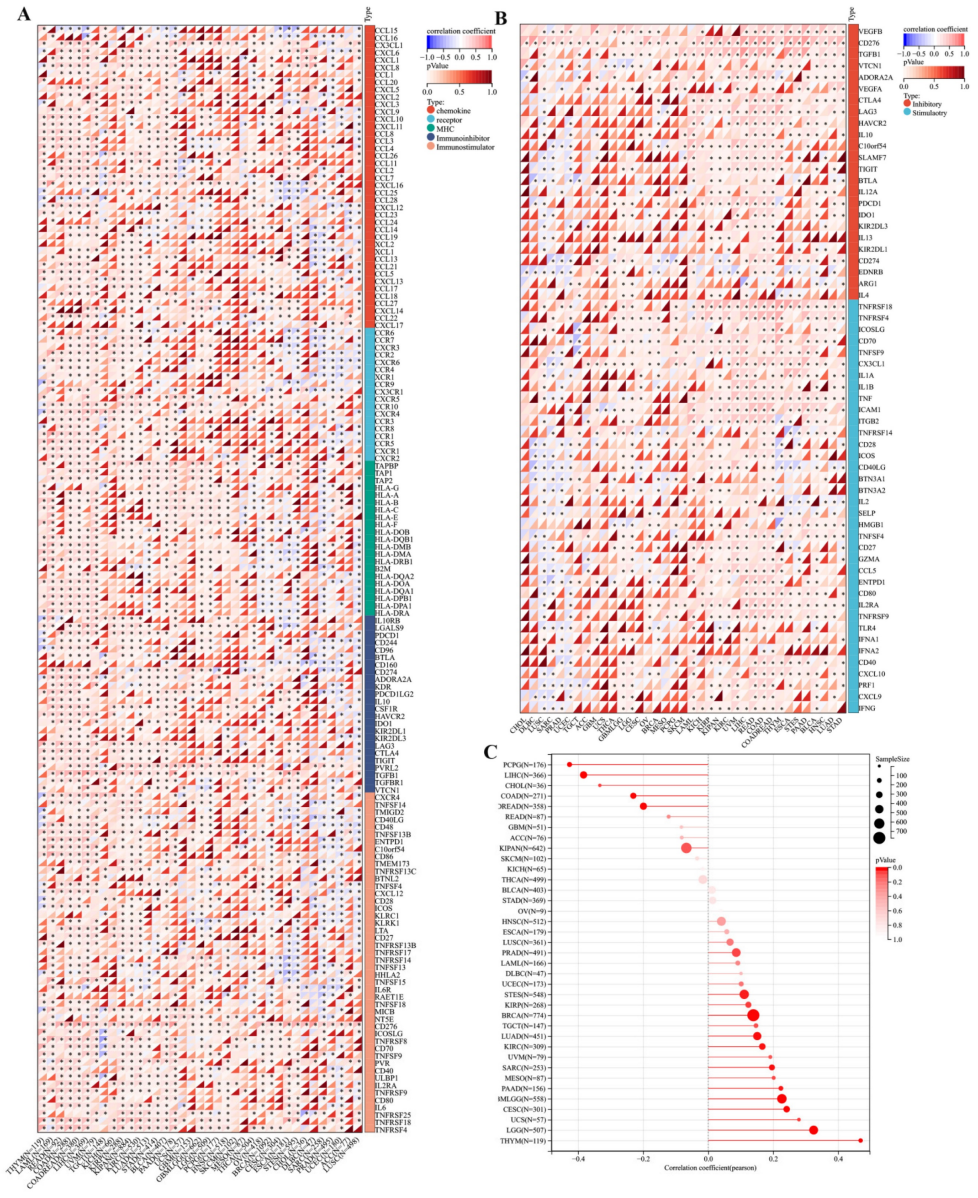
Correlation analysis of TMEM132A and immune regulatory gene, immune checkpoints and tumor stemness score. The correlation of TMEM132A expression with most immune regulatory gene (A). The correlation of TMEM132A and known immune checkpoints across all TCGA cancers (B). The TMEM132A tumor stemness score (C).

We investigated the immunomodulatory role of TMEM132A in tumors by analysing the correlation between immunomodulatory genes, immune checkpoint, tumor stem score, immune infiltration and TMEM132A. First, we are in the pan-cancer dataset (from the UCSE database download), extracted TMEM132A gene and 150 marker genes: five immune pathways (chemokine (41), receptor (18), MHC (21), Immunoinhibitor (24), Immunostimulator (46)), the expression data of marker genes in each sample were further screened and the Pearson correlation of TMEM132A and five immune pathway marker genes was calculated. The expression of TMEM132A was positively correlated with most immunomodulatory genes (Figure 4A). We then analyzed correlations between TMEM132A and certain immune checkpoints (Inhibitory (24), Stimulatory (36)). Based on the data presented we found a significant correlation was found between TMEM132A and most immune checkpoints (Figure 4B). Further, it was found that the TMEM132A gene and tumor stem score was significantly correlated in 17 tumors, of which there was a significant positive correlation in 12 tumors GBMLGG, LGG, CESC, LUAD, BRCA, STES, SARC, KIRP, KIRC, THYM, PAAD, UCS. There was a significant negative correlation among the five tumors COAD, COADREAD, LIHC, PCPG, CHOL (Figure 4C).

**Figure 5:**
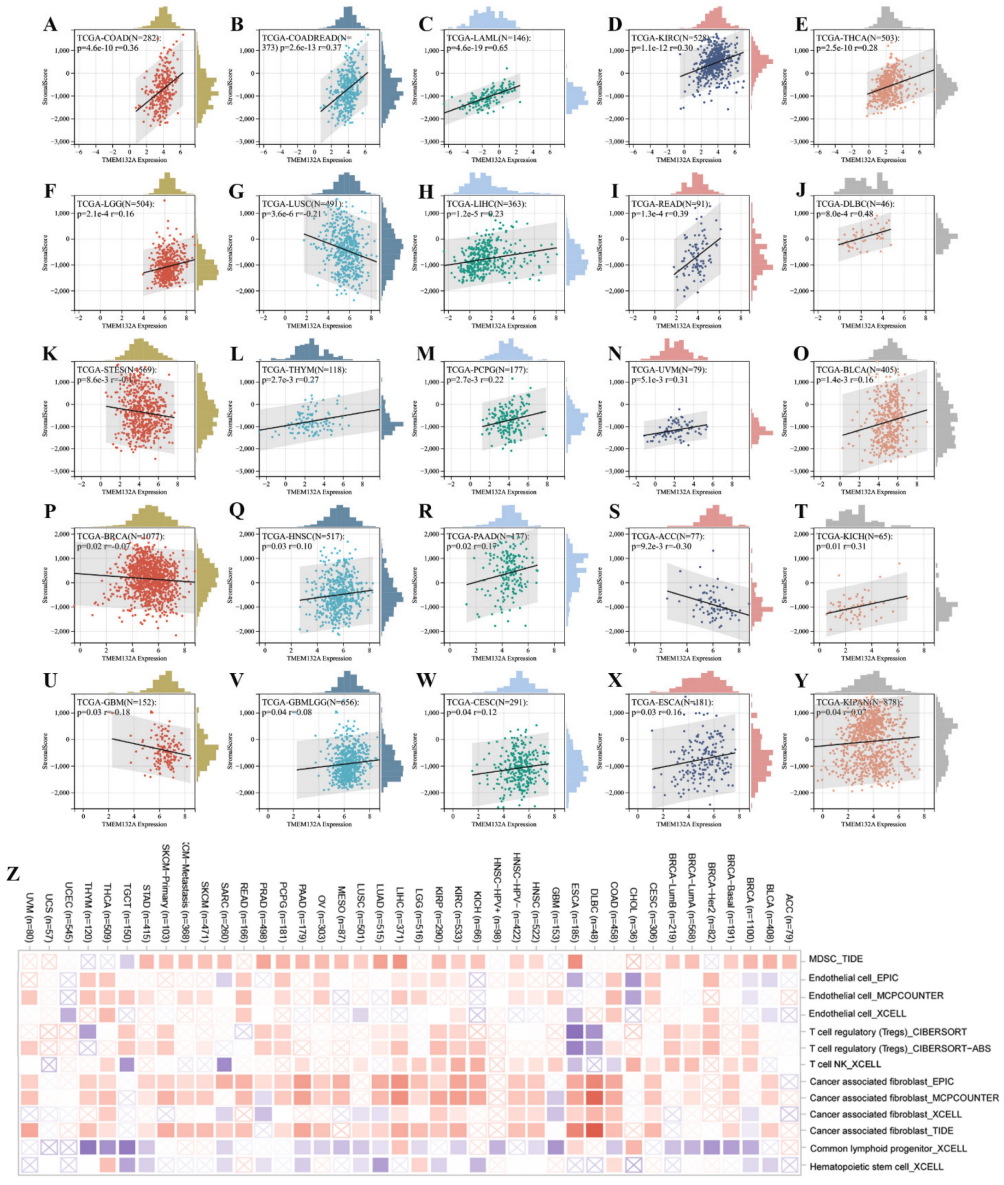
Immune infiltration analysis. Analysis of TMEM132A expression and immune infiltration score in tumor types (A-Y). Examining the correlations between TMEM132A expression and immune infiltration across all TCGA cancers using TIMER2 algorithms (Z).

Furthermore, we found a significant correlation between the expression levels of the TMEM132A gene and tumor immune infiltration scores. Specifically, TMEM132A expression exhibited a significant positive correlation with immune infiltration in 20 types of cancer, including GBMLGG, LGG, CESC, COAD, COADREAD, LAML, ESCA, KIPAN, HNSC, KIRC, THYM, LIHC, THCA, READ, PAAD, PCPG, UVM, BLCA, KICH, and DLBC. Conversely, in 5 other types of cancer, TMEM132A expression showed a significant negative correlation with immune infiltration, including GBM, BRCA, STES, LUSC, and ACC (Figure 5A-Y).

Here, we used TIMER2 to investigate the relationship between TMEM132A expression and the infiltration level of various immune cells in all TCGA cancers. Our study adopted the TIDE, EPIC, MCPCOUNTER, CIBERSORT, CIBERSORT-ABS, MCPCOUNTER, and xCell algorithm algorithms to evaluate the results. The results suggested a positive association between the infiltration level of Myeloid-derived suppressor cells, endothelial, T cell regulator (Treg), neutrophil, T-cell NK, and common associated fibroblast, and a negative correlation was observed between the infiltration level of common lymphoid progenitor, Hematopolrtlc stems cell and TMEM132A expression in multiple tumors (Figure 5Z). This study reveals that the TMEM132A protein may influence tumor immune response by modulating immunomodulatory genes, immune checkpoints, tumor stem scores, or immune infiltration. This research will provide a better understanding of the immune-regulatory mechanisms of tumors and will be crucial in improving the efficacy of immunotherapy.

### 3.4 TMEM132A-related gene analysis

**Figure 6:**
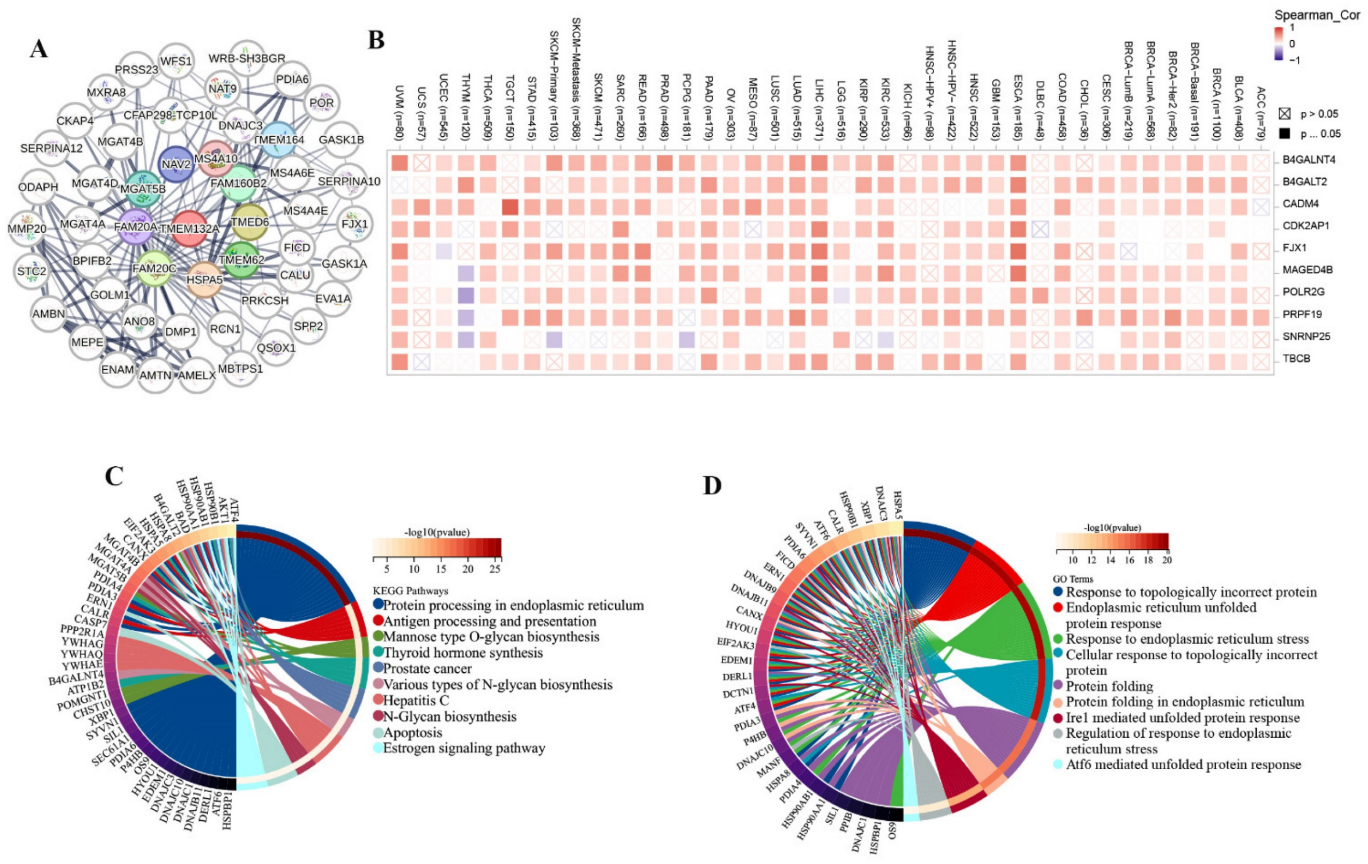
TMEM132A-related gene enrichment analysis. TMEM132A-interacted proteins (A). The expression of top 10 TMEM132A-related targeted genes in cancer (B). KEGG pathway analysis based on the TMEM132A-interacted and correlated genes (C). GO-BP analysis based on the TMEM132A-interacted and correlated genes (D).

We obtained 50 TMEM132A-binding proteins by using the online tool STRING (Figure 6A) (Supplementary Table 1) and identified the top 100 TMEM132A-related targeted genes by using the GEPIA2 tool (Supplementary Table 2). The top 10 TMEM132A-related target genes were positively correlated with tissue-wise expression in different cancer types (Figure 6B). Next, we used the identified genes to perform KEGG and GO enrichment analyzes. KEGG results indicated that TMEM132A might influence tumorigenesis through “Protein processing in endoplasmic reticulum”, “Antigen processing and presentation”, “Mannose type O-glycan biosynthesis” and “Thyroid hormone synthesis”, and other pathways (Figure 6C). GO Biological Processes analysis suggested most of the TMEM132A-related genes were associated with “Response to topologically incorrect protein”, “Endoplasmic reticulum unfolded protein response”, “Response to endoplasmic reticulum stress”, and other functions (Figure 6D).

### 3.5 The characteristics of TMEM132A mutations in the TCGA pan-cancer cohort

**Figure 7:**
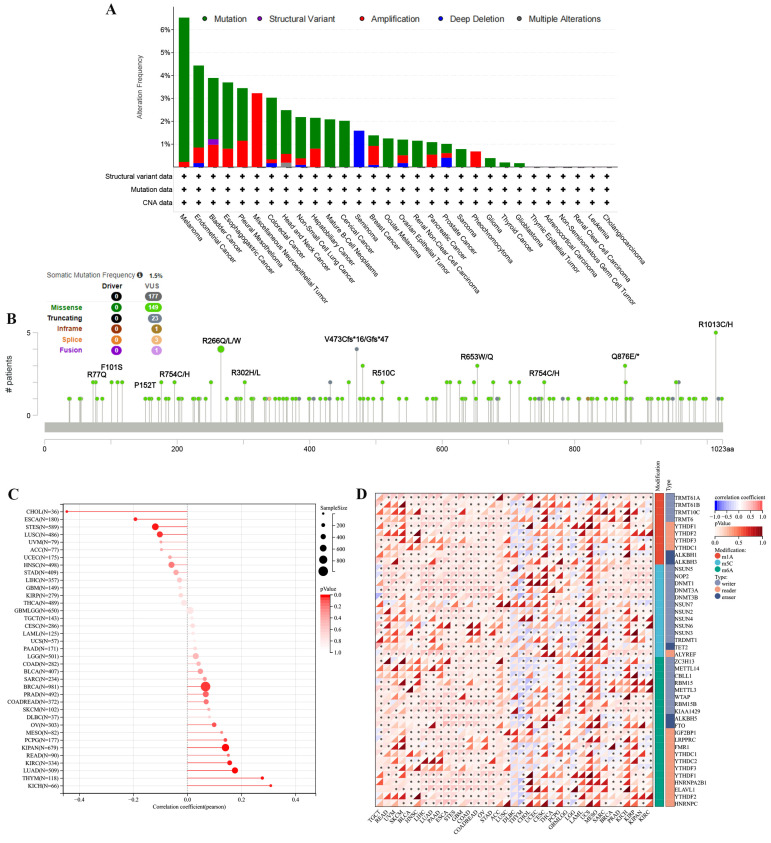
Distinct genomic profiles associated with TMEM132A expression and integrative analysis of complex cancer genomic and clinical profiles. Genetic alteration features (Mutation, Structural Variant, Amplification, and Deep Deletion) of TMEM132A in 32 different tumors were analyzed in the TCGA database by the cBioPortal tool (A); The mutation site of TMEM132A and the number of related cases at this mutation site (B). Genomic heterogeneity and TMEM132A gene expression analysis (C). Expression of the TMEM132A gene and 44 marker genes of class III RNA-modified genes (m1A (10), m5C (13), m6A (21)) in each sample (D).

To investigate whether TMEM132A causes cancer through genetic mutation, we analyzed the mutation sites and types of TMEM132A mutations, RNA modifications, and genomic heterogeneity in pan-cancer. There were 32 cancer types with TMEM132A alteration and Melanoma had the highest frequency of 6.53% (Figure 7A). The results showed the type, locus, and number of cases of TMEM132A gene mutation. The primary form of the genetic mutation was a missense mutation in TMEM132A (Figure 7B) (Supplementary Table 3). In order to better understand the role of TMEM132A in tumors, we analyzed genomic heterogeneity and TMEM132A gene expression: We observed the TMB (Tumor mutation burden) and TMEM132A gene expression data in 37 tumors and showed significant positive correlation in 6 cases (LUAD, BRCA, KIPAN, KIRC, THYM, KICH) and negative correlation in 4 cases (ESCA, STES, LUSC, CHOL) (Figure 7C). To gain greater insight into the molecular mechanisms behind TMEM132A's impact on cancer, we further analyzed the expression of the TMEM132A gene and 44 marker genes of class III RNA-modified genes (m1A (10), m5C (13), m6A (21)) in each sample (Figure 7D). The aforementioned investigation of the TMEM132A gene enhances our comprehension of the molecular process of cancer and proposes novel strategies for the treatment of this disease.

### 3.6 The function analysis of TMEM132A in UCEC, LUAD and LIHC

**Figure 8:**
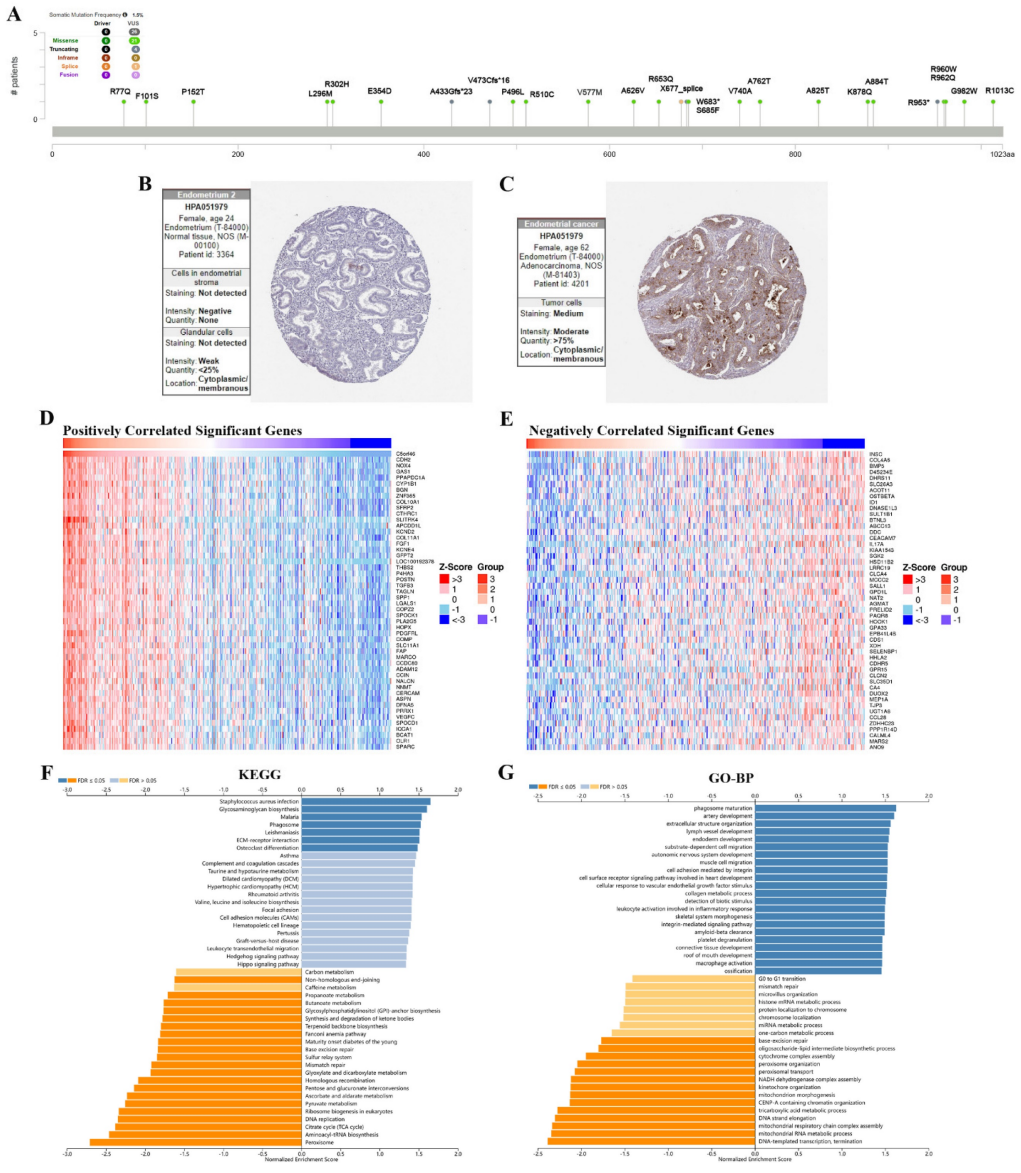
The enrichment analysis of TMEM132A co-expression genes in UCEC. The mutation site of TMEM132A and the number of related cases at this mutation site in UCEC (A). TMEM132A gene expression in Endometrium normal tissues of immunohistochemistry images (B). TMEM132A gene expression in Endometrial cancer tissues of immunohistochemistry images (C). The top 50 genes positively correlated to TMEM132A in UCEC (D). The top 50 genes negatively correlated to TMEM132A in UCEC (E). KEGG analysis of TMEM132A co-expression genes in the UCEC cohort (F). GO analysis of TMEM132A co-expression genes in the UCEC cohort (G).

The results showed the type, locus, and number of cases of TMEM132A gene mutation in UCEC. The primary form of the genetic mutation was a missense mutation in TMEM132A in UCEC (Figure 8A). Immunohistochemical analysis showed higher expression of TMEM132A in Endometrial cancer tissues compared to normal tissues (Figure 8B-C). The top 50 positively related genes with TMEM132A and negatively related genes (Figures 8D-E). Furthermore, KEGG analysis revealed enrichment in *Staphylococcus aureus* infection, Glycosaminoglycan biosynthesis, Malaria, etc. (Figure 8F). GO analysis (biological processes) showed that TMEM132A is mainly related to phagosome maturation, artery development, extracellular structure organization, etc. (Figure 8G).

**Figure 9:**
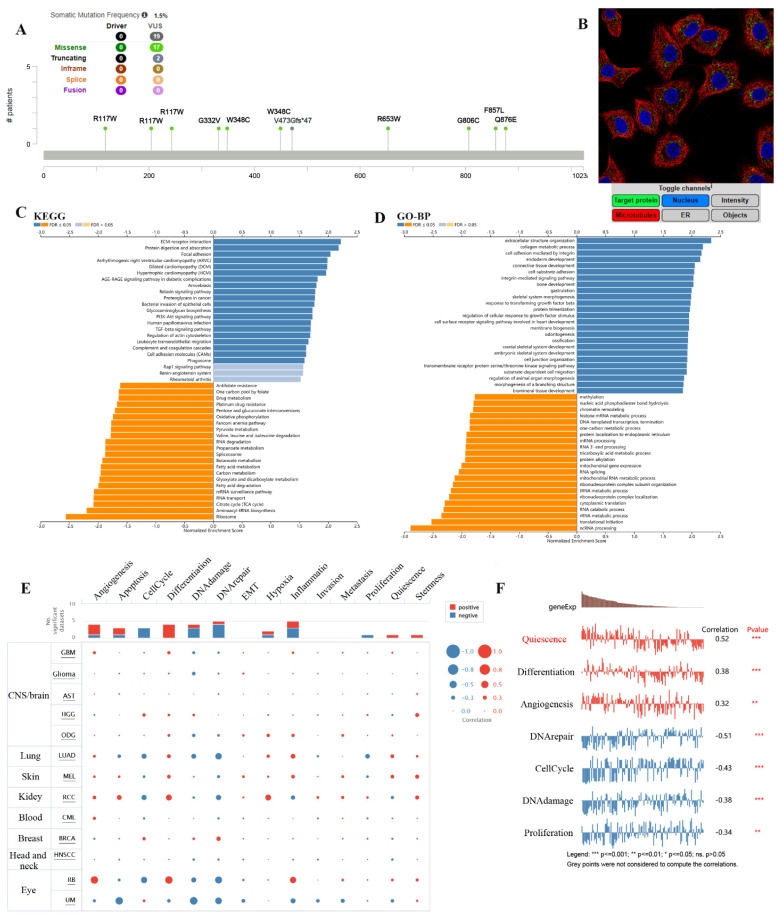
The enrichment analysis of TMEM132A co-expression genes in LUAD. The mutation site of TMEM132A and the number of related cases at this mutation site in LUAD (A). TMEM132A gene expression in A549 of immunofluorescence images (B). KEGG analysis of TMEM132A co-expression genes in the LUAD cohort (C). GO analysis of TMEM132A co-expression genes in the LUAD cohort (D). The correlation found between the expression of TMEM132A and the 14 cancer functional states in pan-cancer was analyzed (E). The expression of TMEM132A correlates with the functional states of LUAD cells. *p < 0.05; **p < 0.01; ***p < 0.001 (F).

The results showed the type, locus, and number of cases of TMEM132A gene mutation in LUAD. The primary form of the genetic mutation was a missense mutation in TMEM132A in LUAD (Figure 9A). Immunofluorescence analysis showed that TMEM132A was expressed in mitochondria of A549 (Non-small cell lung cancer cell lines) (Figure 9B). Furthermore, KEGG analysis revealed enrichment in ECM-receptor interaction, Protein digestion and absorption, Focal adhesion, etc (Figure 9C). GO analysis (biological processes) showed that TMEM132A is mainly related to the extracellular structure organization, collagen metabolic process, cell adhesion mediated by integrin, etc (Figure 9D). We then analyzed the correlation between TMEM132A and 14 cancer functional states using single-cell sequence data from CancerSEA (Figure 9E). As for LUAD, there is a significant positive correlation between TMEM132A expression and Quiescence, Differentiation, and Angiogenesis. And negative correlation between TMEM132A expression and DNA repair, Cell Cycle, DNA damage and Proliferation (Figure 9F).

**Figure 10:**
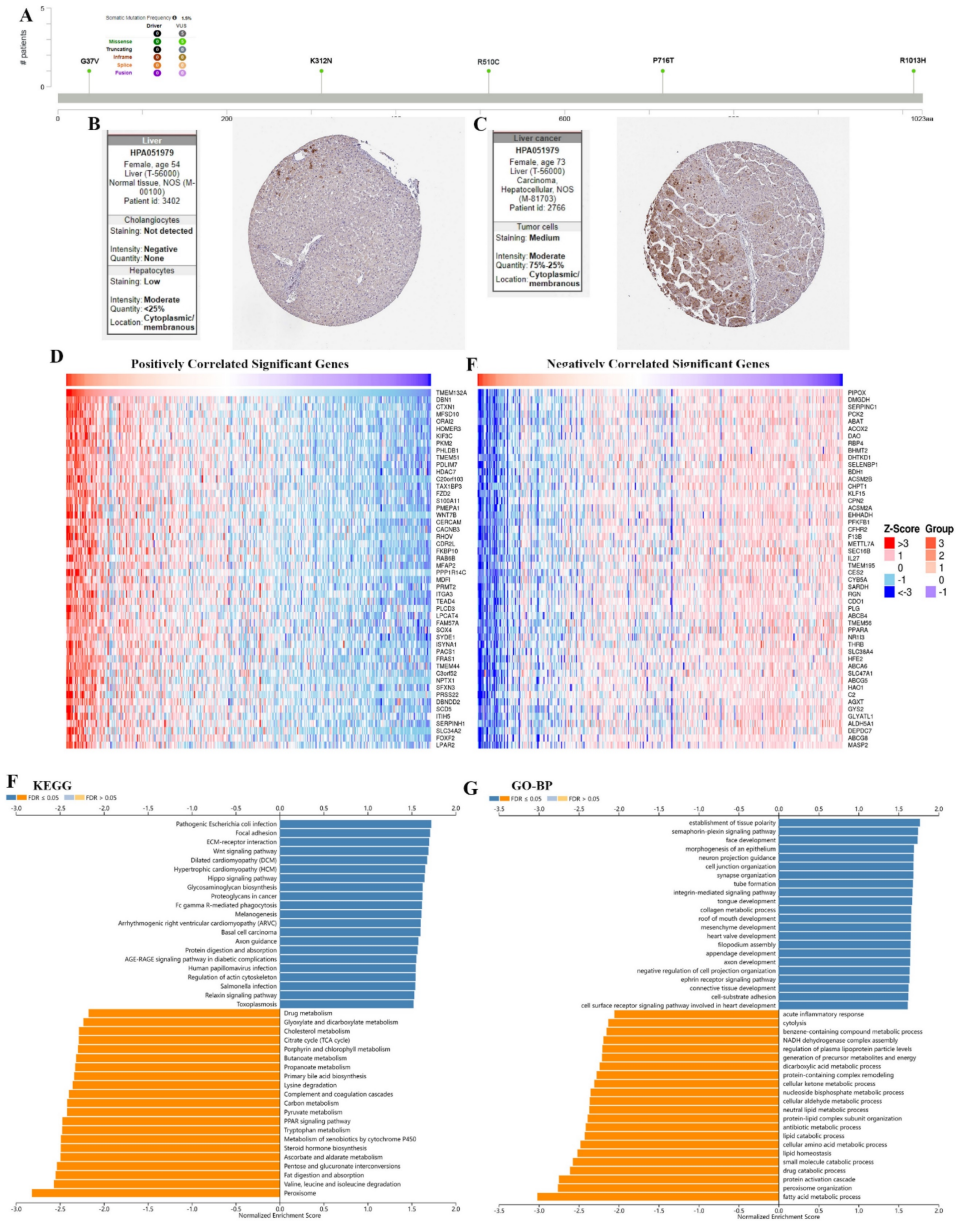
The enrichment analysis of TMEM132A co-expression genes in LIHC. The mutation site of TMEM132A and the number of related cases at this mutation site in LIHC (A). TMEM132A gene expression in Liver normal tissues of immunohistochemistry images (B). TMEM132A gene expression in Liver cancer tissues of immunohistochemistry images (C). The top 50 genes positively correlated to TMEM132A in LIHC (D). The top 50 genes negatively correlated to TMEM132A in LIHC (E). KEGG analysis of TMEM132A co-expression genes in the LIHC cohort (F). GO analysis of TMEM132A co-expression genes in the LIHC cohort (G).

The results showed the type, locus, and number of cases of TMEM132A gene mutation in LIHC. The primary form of the genetic mutation was a missense mutation in TMEM132A in LIHC (Figure 10A). Immunohistochemical analysis showed higher expression of TMEM132A in Endometrial cancer tissues compared to normal tissues (Figure 10B-C). The top 50 positively related genes with TMEM132A and negatively related genes (Figures 10D-E). Furthermore, KEGG analysis revealed enrichment in Pathogenic Escherichia coli infection, Focal adhesion, ECM-receptor interaction, etc. (Figure 10F). GO analysis (biological processes) showed that TMEM132A is mainly related to the establishment of tissue polarity, semaphorin-plexin signaling pathway, face development, etc. (Figure 10G).

### 3.7 The immunotherapy analysis of TMEM132A

**Figure 11:**
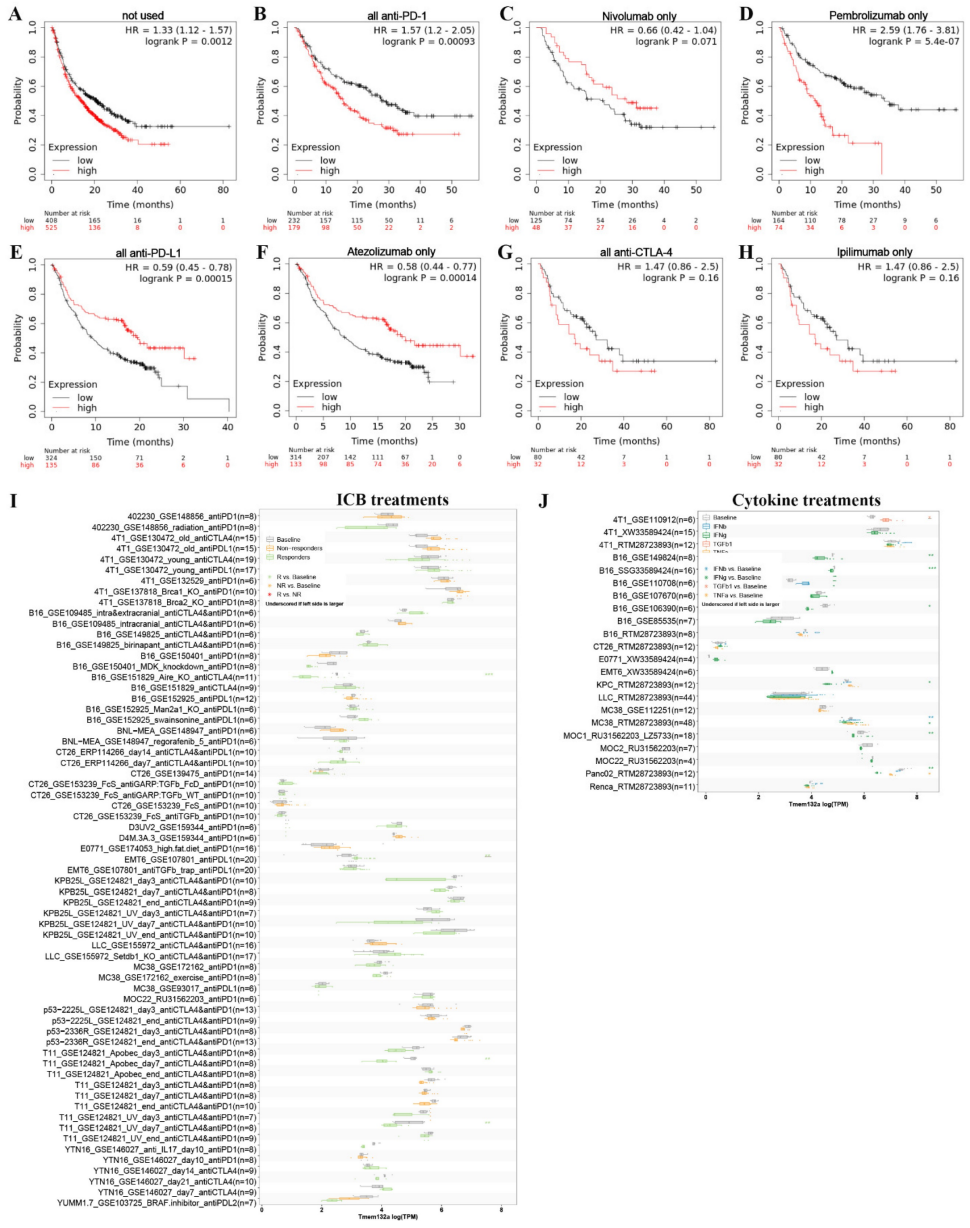
Detection of associations between TMEM132A expression and ICB treatments or Cytokine treatments in cancer. Overall survival without any immunotherapy (A). Overall survival using all anti-PD1 (B). Overall survival using Nivolumab only (anti-PD1) (C). Overall survival using all Pembrolizumab only (anti-PD1) (D). Overall survival using all anti-PD-L1 (E). Overall survival using all Atezolizumab only (anti-PD-L1) (F). Overall survival using all anti-CTLA4 (G). Overall survival using Ipilimumab only (anti-CTLA4) (H). Compare TMEM132A gene expression levels across different tumor models and ICB treatments, between pre- and post-ICB treatment and responders and non-responders (I). Compare TMEM132A gene expression levels across cell-lines between pre- and post-cytokine treated samples (J).

To investigate the potential of TMEM132A as an immunotherapeutic target for tumor treatment, we analyzed the overall survival of patients with high or low expression of TMEM132A after receiving treatment with anti-PD1, anti-PDL1, or anti-CTLA4. The study indicates that patients with high TMEM132A expression had a significantly lower overall survival rate than those with low TMEM132A expression (Figure 11A). Patients who received immunotherapy solely with only Nivolumab (Figure 11C) or all anti-PD-L1 (Figure 11E) or only Atezolizumab (Figure 11F) immunotherapy demonstrated considerably enhanced overall survival with high TMEM132A expression compared to those with low TMEM132A expression. Patients who received only anti-PD1 (Figure 11B) or only Pembrolizumab (Figure 11D) or all anti-CTAL4 (Figure 11G) or only ipilimumab (Figure 11H) immunotherapy with high TMEM132A expression did not improve overall survival compared to patients with low TMEM132A expression. A high level of TMEM132A expression may be associated with a better response to certain immunotherapies, such as anti-PD1, anti-PDL1 or anti-CTLA4. This finding has significant implications for personalised medicine, as it means that by identifying TMEM132A expression levels in patients, physicians can more accurately select appropriate immunotherapies, thereby improving patient survival and treatment efficacy.

Next, we used the Tumor Immune Syngeneic MOuse (TISMO) database to correlate TMEM132A gene expression levels in different tumor models as well as ICB therapy (anti-PD1, anti-PDL1, anti-PDL2, and anti-CTLA4) or cytokine therapy (IFNγ, IFNβ, TNFα, and TGFb1). This analysis aims to gain insight into the potential role and relevance of the TMEM132A gene in cancer therapy. First, the TISMO compares TMEM132A gene expression levels across different tumor models and ICB treatments, between pre- and post-ICB treatment and responders and non-responders. The differences between groups are statistically evaluated by the Wald test using DESeq2 (*FDR ≤ 0.05, **FDR ≤ 0.01, ***FDR ≤ 0.001), and the comparison results are summarized in boxplots. Tumor models included are: Mammary cancer: 4T1, E0771, EMT6, T11, KPB25L, p53-2225L, p53-2336R; Colorectal carcinoma: CT26, MC38; Gastric adenocarcinoma: YTN16; Head and neck squamous cell carcinoma: MOC22; Hepatocellular carcinoma: BNL-MEA; Lung carcinoma: LLC; Melanoma: B16, YUMM1.7, D3UV2, D4M.3A.3; Sarcoma: 402230. Results showed that TMEM132A expression levels were lower among responders compared to baseline in B16_GSE151829_Aire_KO_anti-CTLA4 (n=11), T11_GSE124821_Apobec_day7_anti-CTLA4 & anti-PD1 (n-8), T11_GSE124821_UV_day7_anti-CTLA4 & anti-PD1 (n=8), and with no significant difference in other IBD treatments (Figure 11I). Second, used the TISMO to compare gene expression levels across cell-lines between pre- and post-cytokine treated samples. The differences between groups are statistically evaluated by the Wald test using DESeq2 (*FDR ≤ 0.05, **FDR ≤ 0.01, ***FDR ≤ 0.001), and the comparison results are summarized in boxplots. Cell lines included are: Mammary cancer: 4T1, EMT6, E0771; Colorectal carcinoma: CT26, MC38; Lung carcinoma: LLC; Head and neck squamous cell carcinoma: MOC1; Melanoma: B16; Pancreatic ductal adenocarcinoma: KPC, Panc02; Renal adenocarcinoma: Renca. Results showed that TMEM132A expression levels were lower among IFNγ treatments compared to baseline in 4T1_RTM28723893 (n=6), B16_SSG33589424 (n-16), B16_GSE106390 (n=6), KPC_RTM28723893 (n=6), MC38_RTM28723893 (n=24), MOC1_RU31562203_LZ5733 (n=18), Panc02_RTM28723893 (n=6); TMEM132A expression levels were higher among TGFb1 treatments compared to baseline in 4T1_GSE110912 (n=6); TMEM132A expression levels were lower among IFNβ treatments compared to baseline in MC38 RTM28723893 (n-24); TMEM132A expression levels were lower among TNFα treatments compared to baseline in Panc02_RTM28723893 (n=6). Examining the levels of TMEM132A gene expression in patients receiving ICB or cytokine therapy can ascertain its correlation with the efficacy of immunotherapy. Additionally, it may help identify TMEM132A as a possible biomarker for predicting treatment response. This could enhance our comprehension of TMEM132A's role in cancer treatment and give valuable insights for crafting tailored treatment plans.

## 4. Discussion

Cancer is a condition that results from the abnormal growth and division of cells, whose exact cause may be a complex interaction of various factors 19. To establish the most appropriate treatment plan, a comprehensive analysis of the patient's specific situation, which includes but is not limited to the type of cancer, its grade, the extent of its spread, and the patient's health status, is necessary 20. Advances in science and technology have led to a better understanding of cancer. This has opened up new treatment options. Three areas of interest are ICB treatments, Cytokine treatments and pan-cancer analysis 21, 22, with TMEM132A playing an important role that is also involved in cancer development and treatment.

Immunotherapy represents a major breakthrough in the field of cancer treatment, bringing renewed hope to many patients 23. Despite its promises, however, immunotherapy is beset by obstacles, including inconsistent treatment response and potential side effects, which must be addressed through ongoing research and innovation to fully realise its potential in the fight against cancer 24. Understanding the role of TMEM132A in cancer may offer valuable insights into the disease's underlying mechanisms and establish TMEM132A as an essential biomarker for cancer detection and treatment. This study conducted an extensive bioinformatics analysis of TMEM132A using several public databases. A comprehensive study was undertaken to examine the clinical survival, stage of illness, genetic modifications, correlation with prognosis, immune cell influx, immune checkpoints, Tumor Stemness Score, immunomodulatory factors, genomic patterns, enrichment analyzes, gene groups of interest, immunotherapy, and immune cell function of TMEM132A within the context of human malignancies.

To investigate the role of TMEM132A in pan-cancer, we examined TMEM132A expression in various normal and tumor tissues in humans. We noted that TMEM132A is expressed in a range of tissues, implying it could have a crucial function in diverse physiological and pathological processes. It is noteworthy that TMEM132A is expressed at a higher level in the majority of tumors in comparison to non-tumor tissues, raising the possibility that it plays a significant role in tumor biology. To further explore the important role of TMEM132A expression in tumor diseases, we analyzed the correlation between TMEM132A expression and the clinical stage and prognostic value. The elevated expression level of TMEM132A is linked to the clinical staging of various cancers. In some cancers, high TMEM132A expression has greater prognostic value, indicating that TMEM132A may have a crucial role in tumor development and progression. Subsequent studies have revealed that higher TMEM132A expression is associated with inferior overall and disease-free survival in specific tumor types. This indicates that TMEM132A could function as a promising prognostic indicator, enabling us to forecast patient survival and disease progression. This breakthrough could facilitate the creation of therapeutic methods that focus on TMEM132A, underscoring the importance of TMEM132A immunotherapy in oncology.

The tumor microenvironment (TME) plays a crucial role in the occurrence, growth, and spread of tumors, as well as in the diagnosis, prevention, treatment, and prognosis of cancer 25, 26. It consists of non-cellular components in the extracellular matrix (ECM) and various cellular components, including fibroblasts and immune cells 27, 28. The TME is closely associated with the development, spread, and recurrence of malignant tumors, regulating the proliferation and migration of tumor cells through autocrine-paracrine signaling pathways 29-31. The intricate network relationships within the tumor microenvironment are vital for research centered around immunotherapy targeting the TME 32, 33. The effectiveness of immunotherapy is intricately linked to the status of immune cells within the tumor microenvironment 34, 35, encompassing CD4^+^ T cells, CD8^+^ T cells, myeloid-derived suppressor cells (MDSCs), NKT cells, and others 36, 37. Given the pivotal role of the immune system in cancer, this study delves into the immunological characteristics of TMEM132A in the occurrence and development of tumors. Through an analysis of the relationship between TMEM132A expression and immune regulatory genes, immune checkpoints, tumor stemness scores, immune infiltration, and immune cell infiltration, the study reveals the significant role of TMEM132A within the tumor microenvironment. TMEM132A exhibited significant associations with immune checkpoints, immunomodulators, immunomodulatory genes, tumor stemness score, cell function status, and immune infiltration, indicating its potential role in tumor development through modulation of immune response and invasion. This discovery highlights TMEM132A as a promising candidate for tumor immunotherapy, potentially contributing to improved patient outcomes.

The development of cancer is a complex process involving several factors such as genetics, environment and immune system function 38. Inherited or acquired chromosomal aberrations are frequently connected to human cancers, resulting in the overexpression or repression of genes vital to cell cycle replication, differentiation, and/or apoptosis. Normal cells are transformed into cancer cells due to somatic mutations in critical genes 39. The presence of genomic heterogeneity means that different cancer cells can have different genomic variations, which can cause tumors to have different characteristics and to show different clinical responses in different patients 40-42. An understanding of the types and effects of these variants can help doctors develop better treatment plans that are more effective in responding to the individual differences between patients. In addition, the study of genomic heterogeneity is also contributing to a more in-depth understanding of the biological characteristics of tumors 43. By analysing the distribution and frequency of different variants, researchers can identify potential targeted therapeutic opportunities and develop more targeted drugs 44, 45. This personalised approach to treatment has the potential to improve treatment outcomes, reduce unnecessary side effects and provide patients with a better quality of life. Our previous discussions have highlighted the key role of TMEM132A in the immune response to tumors. In order to gain a better understanding of the role of TMEM132A in tumor development, we analyzed genes and proteins associated with TMEM132A expression and performed a pathway enrichment analysis. Additional analysis has revealed that certain functions and biological processes are enriched in genes linked to TMEM132A, with TMEM132A itself potentially having mutated sites and types. These discoveries offer significant insight for further exploration into the function and mechanisms of TMEM132A, which could lead to a better comprehension of its role in cancer biology.

Immunotherapy medications, namely PD-1, PD-L1, and CTLA-4 inhibitors, exhibit notable effectiveness in multiple cancer types and are presently prevalent in clinical procedures 46. Typically, these remedies are administered as either autonomous therapy alternatives or in conjunction with other therapeutic methods such as chemotherapy and radiation to enhance patient survival rates and quality of life for cancer-afflicted individuals. This treatment approach has not only prolonged patient survival, but has also made substantial strides in enhancing their quality of life 2-5. This study elucidates the potential role and clinical relevance of TMEM132A in tumor immunotherapy by analyzing its expression levels in patients receiving different types of immune treatments. Initially, the study reveals a significant decrease in overall survival rates among patients with high TMEM132A expression, suggesting its potential involvement in tumor progression and immune treatment response. Interestingly, in patients receiving specific immune therapies such as Nivolumab, complete anti-PD-L1, or Atezolizumab, high TMEM132A expression correlates with enhanced overall survival rates, unlike in other immune treatment methods such as pure anti-PD1, pure Pembrolizumab, complete anti-CTAL4, or pure ipilimumab, where no such association is observed. Despite both Nivolumab and Pembrolizumab targeting the PD-1/PD-L1 pathway, the opposite association between high TMEM132A expression and treatment efficacy may be attributed to complex regulatory mechanisms, differences in tumor microenvironments, and variations in individual immune systems. This underscores the importance of considering TMEM132A expression levels and their association with immune therapy when formulating personalized treatment strategies to maximize therapeutic efficacy. Furthermore, further analysis using the TISMO database validates the expression patterns of TMEM132A in different tumor models and treatment regimens. Results indicate a close correlation between TMEM132A expression levels and treatment responsiveness, particularly in anti-CTLA4 therapy. This suggests that TMEM132A may serve as a crucial immunotherapy target, influencing patients' responses to immune treatments in certain therapeutic contexts. Additionally, additional analysis of cell lines demonstrates varying TMEM132A expression levels under different cytokine treatments, implying TMEM132A's role as a regulator of tumor immune responses, influenced by tumor microenvironments and treatment types.

Overall, this in-depth study of cancer emphasises the significance of TMEM132A in the evaluation of prognosis and categorization of cancer, particularly in the area of immunotherapy. Conducting additional research on the mechanisms of TMEM132A is likely to enable us to develop a more precise comprehension of its role in tumor biology and offer inventive therapy alternatives to patients with cancer. The outcomes of this study could potentially have a favorable impact on forthcoming clinical procedures, increasing the prospects of cancer patients. Although the current study provides preliminary insights into the role of the TMEM132A gene in cancer, it is still limited by its reliance on publicly available databases and lack of experimental validation. Future research should integrate various factors including genetics, environment, and the immune system to further elucidate the specific functions and mechanisms of TMEM132A. By establishing animal models and conducting cell experiments, it is anticipated that therapeutic strategies targeting TMEM132A, such as small molecule drugs and gene editing technologies, could be developed, bringing new hope for cancer treatment. Additionally, the expression and mutation status of TMEM132A may contribute to cancer diagnosis and prognosis assessment, driving the development of precision oncology.

## Supplementary Material

Supplementary tables.

## References

[1] Gayet B, Fuks D (2019). Afterword. Surgical oncology clinics of North America.

[2] Baskar R, Lee KA, Yeo R, Yeoh KW (2012). Cancer and radiation therapy: current advances and future directions. International journal of medical sciences.

[3] Pérez-Herrero E, Fernández-Medarde A (2015). Advanced targeted therapies in cancer: Drug nanocarriers, the future of chemotherapy. European journal of pharmaceutics and biopharmaceutics: official journal of Arbeitsgemeinschaft fur Pharmazeutische Verfahrenstechnik eV.

[4] Lv J, Shim JS (2015). Existing drugs and their application in drug discovery targeting cancer stem cells. Archives of pharmacal research.

[5] Zhang Y, Zhang Z (2020). The history and advances in cancer immunotherapy: understanding the characteristics of tumor-infiltrating immune cells and their therapeutic implications. Cellular & molecular immunology.

[6] Pardoll DM (2012). The blockade of immune checkpoints in cancer immunotherapy. Nature reviews Cancer.

[7] Morris EC, Neelapu SS, Giavridis T, Sadelain M (2022). Cytokine release syndrome and associated neurotoxicity in cancer immunotherapy. Nature reviews Immunology.

[8] Priestley P, Baber J, Lolkema MP, Steeghs N, de Bruijn E, Shale C (2019). Pan-cancer whole-genome analyses of metastatic solid tumours. Nature.

[9] Liu J, Lichtenberg T, Hoadley KA, Poisson LM, Lazar AJ, Cherniack AD (2018). An Integrated TCGA Pan-Cancer Clinical Data Resource to Drive High-Quality Survival Outcome Analytics. Cell.

[10] Sanchez-Pulido L, Ponting CP (2018). TMEM132: an ancient architecture of cohesin and immunoglobulin domains define a new family of neural adhesion molecules. Bioinformatics (Oxford, England).

[11] Oh-Hashi K, Sone A, Hikiji T, Hirata Y, Vitiello M, Fedele M (2015). Transcriptional and post-transcriptional regulation of transmembrane protein 132A. Molecular and cellular biochemistry.

[12] Gao Q, Chen Y, Yue L, Li Z, Wang M (2023). Knockdown of TMEM132A restrains the malignant phenotype of gastric cancer cells via inhibiting Wnt signaling. Nucleosides, nucleotides & nucleic acids.

[13] Li B, Niswander LA (2020). TMEM132A, a Novel Wnt Signaling Pathway Regulator Through Wntless (WLS) Interaction. Front Cell Dev Biol.

[14] Oh-hashi K, Imai K, Koga H, Hirata Y, Kiuchi K (2010). Knockdown of transmembrane protein 132A by RNA interference facilitates serum starvation-induced cell death in Neuro2a cells. Molecular and cellular biochemistry.

[15] Li B, Brusman L, Dahlka J, Niswander LA (2022). TMEM132A ensures mouse caudal neural tube closure and regulates integrin-based mesodermal migration. Development.

[16] Zeng H, Liu A (2023). TMEM132A regulates mouse hindgut morphogenesis and caudal development. Development.

[17] Wu R, Li D, Feng D, Han P (2023). Transmembrane protein 132A (TMEM132A) predicts overall survival for bladder cancer patients. Asian journal of surgery.

[18] Shen W, Song Z, Zhong X, Huang M, Shen D, Gao P (2022). Sangerbox: a comprehensive, interaction-friendly clinical bioinformatics analysis platform. J Imeta.

[19] Fadaka A, Ajiboye B, Ojo O, Adewale O, Olayide I, Emuowhochere RJJoOS (2017). Biology of glucose metabolization in cancer cells. J Onc Sci.

[20] Beutler LE, Someah K, Kimpara S, Miller K (2016). Selecting the most appropriate treatment for each patient. International journal of clinical and health psychology: IJCHP.

[21] Rallis KS, Corrigan AE, Dadah H, George AM, Keshwara SM, Sideris M (2021). Cytokine-based Cancer Immunotherapy: Challenges and Opportunities for IL-10. Anticancer research.

[22] Zhou X, Ni Y, Liang X, Lin Y, An B, He X (2022). Mechanisms of tumor resistance to immune checkpoint blockade and combination strategies to overcome resistance. Frontiers in immunology.

[23] Waldman AD, Fritz JM, Lenardo MJ (2020). A guide to cancer immunotherapy: from T cell basic science to clinical practice. Nature reviews Immunology.

[24] Gupta S, Shukla S (2022). Limitations of Immunotherapy in Cancer. Cureus.

[25] Gong H, Chao Y, Xiang J, Han X, Song G, Feng L (2016). Hyaluronidase To Enhance Nanoparticle-Based Photodynamic Tumor Therapy. Nano letters.

[26] Liu C, Wang M, Zhang H, Li C, Zhang T, Liu H (2022). Tumor microenvironment and immunotherapy of oral cancer. European journal of medical research.

[27] Wu J, Chen J, Feng Y, Zhang S, Lin L, Guo Z (2020). An immune cocktail therapy to realize multiple boosting of the cancer-immunity cycle by combination of drug/gene delivery nanoparticles. Science advances.

[28] Sharma P, Hu-Lieskovan S, Wargo JA, Ribas A (2017). Primary, Adaptive, and Acquired Resistance to Cancer Immunotherapy. Cell.

[29] McNutt M (2013). Cancer immunotherapy. Science (New York, NY).

[30] Guan X, Lin L, Chen J, Hu Y, Sun P, Tian H (2019). Efficient PD-L1 gene silence promoted by hyaluronidase for cancer immunotherapy. Journal of controlled release: official journal of the Controlled Release Society.

[31] Luo Z, Wang Q, Lau WB, Lau B, Xu L, Zhao L (2016). Tumor microenvironment: The culprit for ovarian cancer metastasis?. Cancer letters.

[32] Yang J, Yang Y, Kawazoe N, Chen G (2019). Encapsulation of individual living cells with enzyme responsive polymer nanoshell. Biomaterials.

[33] Chauhan VP, Stylianopoulos T, Martin JD, Popović Z, Chen O, Kamoun WS (2012). Normalization of tumour blood vessels improves the delivery of nanomedicines in a size-dependent manner. Nature nanotechnology.

[34] Yu X, Shi S, Yu X (2022). Immune microenvironment and immunotherapy in pancreatic cancer: a narrative review. Precis Cancer Med.

[35] Zhang J, Shi Z, Xu X, Yu Z, Mi J (2019). The influence of microenvironment on tumor immunotherapy. The FEBS journal.

[36] Oura K, Morishita A, Tani J, Masaki T (2021). Tumor Immune Microenvironment and Immunosuppressive Therapy in Hepatocellular Carcinoma: A Review. International journal of molecular sciences.

[37] Hinshaw DC, Shevde LA (2019). The Tumor Microenvironment Innately Modulates Cancer Progression. Cancer research.

[38] Pandya PH, Murray ME, Pollok KE, Renbarger JL (2016). The Immune System in Cancer Pathogenesis: Potential Therapeutic Approaches. Journal of immunology research.

[39] Martínez-Jiménez F, Muiños F, Sentís I, Deu-Pons J, Reyes-Salazar I, Arnedo-Pac C (2020). A compendium of mutational cancer driver genes. Nature reviews Cancer.

[40] Malone ER, Oliva M, Sabatini PJB, Stockley TL, Siu LL (2020). Molecular profiling for precision cancer therapies. Genome medicine.

[41] Zhang J, Späth SS, Marjani SL, Zhang W, Pan X (2018). Characterization of cancer genomic heterogeneity by next-generation sequencing advances precision medicine in cancer treatment. Precision clinical medicine.

[42] Fisher R, Pusztai L, Swanton C (2013). Cancer heterogeneity: implications for targeted therapeutics. British journal of cancer.

[43] McQuerry JA, Chang JT, Bowtell DDL, Cohen A, Bild AH (2017). Mechanisms and clinical implications of tumor heterogeneity and convergence on recurrent phenotypes. Journal of molecular medicine (Berlin, Germany).

[44] Saunders NA, Simpson F, Thompson EW, Hill MM, Endo-Munoz L, Leggatt G (2012). Role of intratumoural heterogeneity in cancer drug resistance: molecular and clinical perspectives. EMBO molecular medicine.

[45] Laajala TD, Gerke T, Tyekucheva S, Costello JC (2019). Modeling genetic heterogeneity of drug response and resistance in cancer. Current opinion in systems biology.

[46] Shiravand Y, Khodadadi F, Kashani SMA, Hosseini-Fard SR, Hosseini S, Sadeghirad H (2022). Immune Checkpoint Inhibitors in Cancer Therapy. Current oncology (Toronto, Ont).

