# Supplementary material for: Multidimensional analysis of TMEM132A in pan-cancer: unveiling its potential as a biomarker for treatment response prediction
Source: J Cancer. 2024 Jun 11;15(13):4386–405. doi: 10.7150/jca.96396 (PMC11212083; doi:10.7150/jca.96396)
Supplement: Supplementary file 1 — Supplementary tables. [file jcav15p4386s1.pdf]

Supplementary Table 1

#node	identifier	node_degree
A2M	9606.ENSP00000323929	2
AKT1	9606.ENSP00000451828	18
ATF4	9606.ENSP00000336790	16
ATF6	9606.ENSP00000356919	31
CALR	9606.ENSP00000320866	29
CANX	9606.ENSP00000247461	31
CASP7	9606.ENSP00000358327	7
DERL1	9606.ENSP00000259512	20
DNAJB11	9606.ENSP00000414398	25
DNAJB9	9606.ENSP00000249356	26
DNAJC1	9606.ENSP00000366179	8
DNAJC10	9606.ENSP00000264065	24
DNAJC3	9606.ENSP00000473631	30
EDEM1	9606.ENSP00000256497	24
EIF2AK3	9606.ENSP00000307235	20
ERN1	9606.ENSP00000401445	28
FAM160B2	9606.ENSP00000289921	1
FAM20A	9606.ENSP00000468308	7
FAM20C	9606.ENSP00000322323	7
FICD	9606.ENSP00000446479	1
GPX4	9606.ENSP00000346103	4
HSP90AA1	9606.ENSP00000335153	29
HSP90AB1	9606.ENSP00000360609	27
HSP90B1	9606.ENSP00000299767	34
HSPA5	9606.ENSP00000324173	38
HSPA8	9606.ENSP00000437125	25
HYOU1	9606.ENSP00000480150	30
MANF	9606.ENSP00000499582	17
MGAT4A	9606.ENSP00000264968	5
MGAT4B	9606.ENSP00000338487	3
MGAT5B	9606.ENSP00000391227	5
MS4A10	9606.ENSP00000311862	1
NAV2	9606.ENSP00000379396	2
OS9	9606.ENSP00000318165	22
P4HB	9606.ENSP00000327801	28
PDIA3	9606.ENSP00000300289	27
PDIA4	9606.ENSP00000499129	27
PDIA6	9606.ENSP00000385385	26
POMGNT1	9606.ENSP00000361060	3
PPIB	9606.ENSP00000300026	16
PRNP	9606.ENSP00000399376	9
SEC61A1	9606.ENSP00000243253	24
SIGMAR1	9606.ENSP00000277010	7
SIL1	9606.ENSP00000265195	18
SOD1	9606.ENSP00000270142	17
SYVN1	9606.ENSP00000366395	18
TMED6	9606.ENSP00000288025	1
TMEM132A	9606.ENSP00000005286	10
TMEM164	9606.ENSP00000361138	2
TMEM62	9606.ENSP00000260403	2
XBP1	9606.ENSP00000216037	30

Supplementary Table 2

Gene Symbol	Gene ID	PCC	Gene Symbol	Gene ID	PCC
B4GALNT4	ENSG00000182272.11	0.49	AC092171.4	ENSG00000230733.2	0.36
B4GALT2	ENSG00000117411.16	0.44	PSMB5	ENSG00000100804.18	0.36
PRPF19	ENSG00000110107.8	0.44	AP2M1	ENSG00000161203.13	0.36
CDK2AP1	ENSG00000111328.6	0.43	OLFM2	ENSG00000105088.8	0.36
TBCB	ENSG00000105254.11	0.42	TMEM206	ENSG00000065600.12	0.36
POLR2G	ENSG00000168002.11	0.41	VPS72	ENSG00000163159.11	0.36
CADM4	ENSG00000105767.2	0.4	BANF1	ENSG00000175334.7	0.36
SNRNP25	ENSG00000161981.10	0.4	C1orf122	ENSG00000197982.13	0.35
FJX1	ENSG00000179431.6	0.4	DYNC1H1	ENSG00000197102.10	0.35
MAGED4B	ENSG00000187243.16	0.39	LLGL1	ENSG00000131899.10	0.35
GNA12	ENSG00000146535.13	0.39	PRCC	ENSG00000143294.14	0.35
CLSTN1	ENSG00000171603.16	0.39	GDF1	ENSG00000130283.8	0.35
METRN	ENSG00000103260.8	0.39	SFXN5	ENSG00000144040.12	0.35
ENOPH1	ENSG00000145293.14	0.39	FAM89B	ENSG00000176973.7	0.35
PAFAH1B3	ENSG00000079462.7	0.39	ANAPC11	ENSG00000141552.17	0.35
MLF2	ENSG00000089693.10	0.38	MARCKSL1	ENSG00000175130.6	0.35
YWHAE	ENSG00000108953.16	0.38	ERAL1	ENSG00000132591.11	0.35
ERI3	ENSG00000117419.14	0.38	MARCKS	ENSG00000277443.1	0.35
CDK5	ENSG00000164885.12	0.38	YWHAG	ENSG00000170027.6	0.35
DYNLL1	ENSG00000088986.10	0.38	ALKBH4	ENSG00000160993.3	0.35
POP7	ENSG00000172336.4	0.38	RNF187	ENSG00000168159.10	0.35
SMOX	ENSG00000088826.17	0.38	B3GALT6	ENSG00000176022.4	0.35
RP11-881M11.4	ENSG00000256196.1	0.38	HSPBP1	ENSG00000133265.10	0.35
STX6	ENSG00000135823.13	0.38	GPR137C	ENSG00000180998.11	0.35
PAQR4	ENSG00000162073.13	0.38	MRPL28	ENSG00000086504.15	0.35
TUBB3	ENSG00000258947.6	0.38	CHST10	ENSG00000115526.10	0.35
YKT6	ENSG00000106636.7	0.37	CENPB	ENSG00000125817.7	0.35
PFN2	ENSG00000070087.13	0.37	TSC22D4	ENSG00000166925.8	0.35
DDR1	ENSG00000204580.11	0.37	PSMC3	ENSG00000165916.8	0.35
NRSN2	ENSG00000125841.12	0.37	MYEOV2	ENSG00000172428.10	0.35
PSMG3	ENSG00000157778.8	0.37	ZNF668	ENSG00000167394.12	0.35
APBA2	ENSG00000034053.14	0.37	DDX49	ENSG00000105671.11	0.35
ATP1B2	ENSG00000129244.8	0.37	FSCN1	ENSG00000075618.17	0.35
PPP1R14B	ENSG00000173457.10	0.37	APOO	ENSG00000184831.13	0.35
CERS1	ENSG00000223802.7	0.37	ADGRB2	ENSG00000121753.12	0.35
ARF5	ENSG00000004059.10	0.37	PDZD11	ENSG00000120509.10	0.35
FEZ1	ENSG00000149557.12	0.37	PPP2R1A	ENSG00000105568.17	0.34
VAX2	ENSG00000116035.2	0.37	SNN	ENSG00000184602.5	0.34
NABP2	ENSG00000139579.12	0.36	PUF60	ENSG00000179950.13	0.34
DCTN1	ENSG00000204843.12	0.36	SMIM13	ENSG00000224531.5	0.34
OTUB1	ENSG00000167770.11	0.36	ZNF428	ENSG00000131116.11	0.34
HS6ST1	ENSG00000136720.6	0.36	TSSC1	ENSG00000032389.12	0.34
LIMK1	ENSG00000106683.14	0.36	DPF1	ENSG00000011332.19	0.34
CBX1	ENSG00000108468.14	0.36	CCDC167	ENSG00000198937.8	0.34
NCBP2	ENSG00000114503.10	0.36	SMARCA4	ENSG00000127616.17	0.34
TUBB2B	ENSG00000137285.9	0.36	TRIM47	ENSG00000132481.6	0.34
SLC1A3	ENSG00000079215.13	0.36	BAD	ENSG00000002330.13	0.34
FIBP	ENSG00000172500.12	0.36	GPR37L1	ENSG00000170075.8	0.34
USP5	ENSG00000111667.13	0.36	DBN1	ENSG00000113758.13	0.34
YWHAQ	ENSG00000134308.13	0.36	WSB2	ENSG00000176871.8	0.34

Supplementary Table 3

Cancer Type Acronym	Sample ID	**Protein Change**	Mutation Type
LGG	TCGA-DU-6392-01	**R480C**	Missense_Mutation
LGG	TCGA-DB-A64X-01	**R315C**	Missense_Mutation
BLCA	TCGA-FD-A3SJ-01	**R161W**	Missense_Mutation
BLCA	TCGA-FD-A3B5-01	**R110***	Nonsense_Mutation
BLCA	TCGA-DK-A6AW-01	**R77Q**	Missense_Mutation
BLCA	TCGA-FD-A6TC-01	**Q876E**	Missense_Mutation
BLCA	TCGA-H4-A2HQ-01	**Q876***	Nonsense_Mutation
BLCA	TCGA-K4-A54R-01	**S196L**	Missense_Mutation
BLCA	TCGA-XF-A9T8-01	**D54H**	Missense_Mutation
BLCA	TCGA-ZF-AA4N-01	**F101L**	Missense_Mutation
BLCA	TCGA-DK-A3WW-01	**P374R**	Missense_Mutation
BLCA	TCGA-GV-A3JZ-01	**E612K**	Missense_Mutation
BLCA	TCGA-XF-A9SZ-01	**R749C**	Missense_Mutation
BLCA	TCGA-FD-A62N-01	**LIPA-TMEM132A Fusion**	fusion
BRCA	TCGA-EW-A2FV-01	**T888Pfs*18**	Frame_Shift_Del
BRCA	TCGA-AC-A23H-01	**S73C**	Missense_Mutation
BRCA	TCGA-AO-A128-01	**T630M**	Missense_Mutation
BRCA	TCGA-LL-A7SZ-01	**E406***	Nonsense_Mutation
BRCA	TCGA-B6-A0IP-01	**P366L**	Missense_Mutation
CESC	TCGA-VS-A958-01	**S73C**	Missense_Mutation
CESC	TCGA-DR-A0ZM-01	**L651F**	Missense_Mutation
CESC	TCGA-EA-A1QT-01	**R754C**	Missense_Mutation
CESC	TCGA-VS-A9U6-01	**A673T**	Missense_Mutation
CESC	TCGA-2W-A8YY-01	**A933V**	Missense_Mutation
CESC	TCGA-JW-A5VL-01	**S635L**	Missense_Mutation
COAD	TCGA-AA-3977-01	**R480C**	Missense_Mutation
COAD	TCGA-AA-A02O-01	**R911Q**	Missense_Mutation
COAD	TCGA-AZ-4315-01	**R266Q**	Missense_Mutation
COAD	TCGA-A6-2686-01	**A433Pfs*5**	Frame_Shift_Del
COAD	TCGA-AZ-6598-01	**V473Cfs*16**	Frame_Shift_Del
COAD	TCGA-CK-6746-01	**R959Gfs*5**	Frame_Shift_Del
COAD	TCGA-CM-6171-01	**S648L**	Missense_Mutation
COAD	TCGA-A6-5661-01	**R266W**	Missense_Mutation
COAD	TCGA-AA-3715-01	**R266L**	Missense_Mutation
COAD	TCGA-F4-6703-01	**Q38H**	Missense_Mutation
SKCM	TCGA-GF-A3OT-06	**R266Q**	Missense_Mutation
SKCM	TCGA-FW-A3R5-06	**P233S**	Missense_Mutation
SKCM	TCGA-FW-A3R5-06	**R110Q**	Missense_Mutation
SKCM	TCGA-FW-A3R5-06	**S648L**	Missense_Mutation
SKCM	TCGA-D3-A8GM-06	**G236Vfs*21**	Frame_Shift_Del
SKCM	TCGA-DA-A1I1-06	**L488V**	Missense_Mutation
SKCM	TCGA-EB-A3XC-01	**P914S**	Missense_Mutation
SKCM	TCGA-EE-A181-06	**T790I**	Missense_Mutation
SKCM	TCGA-EE-A20H-06	**S1022R**	Missense_Mutation
SKCM	TCGA-EE-A29E-06	**A459V**	Missense_Mutation
SKCM	TCGA-EE-A2A1-06	**A459V**	Missense_Mutation
SKCM	TCGA-EE-A2GU-06	**P768L**	Missense_Mutation
SKCM	TCGA-EE-A2MC-06	**E834K**	Missense_Mutation
SKCM	TCGA-EE-A2MD-06	**E926K**	Missense_Mutation
SKCM	TCGA-EE-A2MR-06	**L399F**	Missense_Mutation
SKCM	TCGA-EE-A3JI-06	**E335Afs*7**	Frame_Shift_Del
SKCM	TCGA-ER-A194-01	**P941S**	Missense_Mutation

SKCM	TCGA-FW-A3R5-06	**E379K**	Missense_Mutation
SKCM	TCGA-FW-A3R5-06	**R750W**	Missense_Mutation
SKCM	TCGA-GN-A266-06	**G416R**	Missense_Mutation
SKCM	TCGA-GN-A26C-01	**E677K**	Missense_Mutation
SKCM	TCGA-D3-A2JC-06	**L275I**	Missense_Mutation
SKCM	TCGA-D3-A2JG-06	**P980T**	Missense_Mutation
SKCM	TCGA-D3-A2JL-06	**H172N**	Missense_Mutation
SKCM	TCGA-EE-A2MG-06	**G703***	Nonsense_Mutation
SKCM	TCGA-ER-A194-01	**G942E**	Missense_Mutation
SKCM	TCGA-ER-A19C-06	**E1017***	Nonsense_Mutation
SKCM	TCGA-FR-A726-01	**P973L**	Missense_Mutation
SKCM	TCGA-FS-A1ZZ-06	**X612_splice**	Splice_Region
SKCM	TCGA-FW-A3R5-06	**P251L**	Missense_Mutation
SKCM	TCGA-GN-A8LK-06	**P251L**	Missense_Mutation
SKCM	TCGA-W3-AA1O-06	**E827K**	Missense_Mutation
SKCM	TCGA-WE-A8K5-06	**G703E**	Missense_Mutation
SKCM	TCGA-YD-A9TA-06	**S590L**	Missense_Mutation
DLBC	TCGA-FA-A6HN-01	**I400T**	Missense_Mutation
STAD	TCGA-HU-A4H0-01	**A626T**	Missense_Mutation
ESCA	TCGA-L5-A4OF-01	**L53V**	Missense_Mutation
ESCA	TCGA-L5-A4OI-01	**P916L**	Missense_Mutation
GBM	TCGA-06-5416-01	**R591C**	Missense_Mutation
HNSC	TCGA-BA-A6DJ-01	**R176G**	Missense_Mutation
HNSC	TCGA-BA-5153-01	**Y207N**	Missense_Mutation
HNSC	TCGA-H7-A6C5-01	**D226N**	Missense_Mutation
HNSC	TCGA-HD-A634-01	**R302L**	Missense_Mutation
HNSC	TCGA-D6-6516-01	**L232F**	Missense_Mutation
HNSC	TCGA-CV-A45Q-01	**E79Q**	Missense_Mutation
HNSC	TCGA-CV-7440-01	**G360C**	Missense_Mutation
HNSC	TCGA-D6-8568-01	**I863F**	Missense_Mutation
HNSC	TCGA-HD-A4C1-01	**E847Q**	Missense_Mutation
HNSC	TCGA-HD-A6I0-01	**D632N**	Missense_Mutation
HNSC	TCGA-MT-A51X-01	**X339_splice**	Splice_Region
LIHC	TCGA-CC-A5UE-01	**K312N**	Missense_Mutation
LIHC	TCGA-CC-A3MB-01	**R510C**	Missense_Mutation
LIHC	TCGA-DD-AADO-01	**R1013H**	Missense_Mutation
LIHC	TCGA-DD-AADM-01	**P716T**	Missense_Mutation
LIHC	TCGA-GJ-A6C0-01	**G37V**	Missense_Mutation
STAD	TCGA-CG-5728-01	**R1013H**	Missense_Mutation
STAD	TCGA-VQ-A91D-01	**R117Q**	Missense_Mutation
STAD	TCGA-B7-A5TJ-01	**R481Q**	Missense_Mutation
SARC	TCGA-3B-A9HT-01	**R586H**	Missense_Mutation
LUAD	TCGA-05-4432-01	**Q876E**	Missense_Mutation
LUAD	TCGA-55-8207-01	**D243Y**	Missense_Mutation
LUAD	TCGA-62-A46O-01	**G332V**	Missense_Mutation
LUAD	TCGA-67-3771-01	**Q449H**	Missense_Mutation
LUAD	TCGA-86-8073-01	**E204D**	Missense_Mutation
LUAD	TCGA-86-8672-01	**R653W**	Missense_Mutation
LUAD	TCGA-17-Z051-01	**V473Gfs*47**	Frame_Shift_Ins
LUAD	TCGA-50-5045-01	**W348C**	Missense_Mutation
LUAD	TCGA-62-8398-01	**R117W**	Missense_Mutation
LUAD	TCGA-78-8640-01	**F857L**	Missense_Mutation
LUAD	TCGA-91-6829-01	**G806C**	Missense_Mutation
LUSC	TCGA-52-7809-01	**E224Q**	Missense_Mutation

LUSC	TCGA-56-7222-01	**G607S**	Missense_Mutation
LUSC	TCGA-56-A49D-01	**W87R**	Missense_Mutation
LUSC	TCGA-60-2698-01	**S948***	Nonsense_Mutation
LUSC	TCGA-46-3765-01	**R202L**	Missense_Mutation
LUSC	TCGA-46-3768-01	**D910H**	Missense_Mutation
LUSC	TCGA-66-2768-01	**R365L**	Missense_Mutation
LUSC	TCGA-77-6844-01	**G442S**	Missense_Mutation
COAD	TCGA-AA-3821-01	**R480C**	Missense_Mutation
COAD	TCGA-CA-6717-01	**R424C**	Missense_Mutation
COAD	TCGA-A6-3809-01	**D314G**	Missense_Mutation
COAD	TCGA-CK-4951-01	**A55T**	Missense_Mutation
COAD	TCGA-CK-4951-01	**R176H**	Missense_Mutation
COAD	TCGA-D5-6928-01	**A670T**	Missense_Mutation
COAD	TCGA-D5-6928-01	**R877H**	Missense_Mutation
STAD	TCGA-HF-7132-01	**R877C**	Missense_Mutation
PAAD	TCGA-IB-7651-01	**E187D**	Missense_Mutation
PAAD	TCGA-IB-7651-01	**I290V**	Missense_Mutation
KIRP	TCGA-4A-A93X-01	**E746***	Nonsense_Mutation
KIRP	TCGA-BQ-5876-01	**A183P**	Missense_Mutation
KIRP	TCGA-IA-A83S-01	**R998P**	Missense_Mutation
KIRP	TCGA-WN-AB4C-01	**A546T**	Missense_Mutation
STAD	TCGA-VQ-A8PB-01	**M385Wfs*25**	Frame_Shift_Del
THCA	TCGA-EM-A22M-01	**R289L**	Missense_Mutation
MESO	TCGA-LK-A4NY-01	**E994K**	Missense_Mutation
MESO	TCGA-3H-AB3S-01	**R535L**	Missense_Mutation
PRAD	TCGA-V1-A8MG-01	**R1013C**	Missense_Mutation
PRAD	TCGA-KK-A6E0-01	**P674S**	Missense_Mutation
READ	TCGA-AG-A002-01	**R1013C**	Missense_Mutation
OV	TCGA-04-1331-01	**P716R**	Missense_Mutation
OV	TCGA-13-0807-01	**D476N**	Missense_Mutation
OV	TCGA-13-1494-01	**V509F**	Missense_Mutation
OV	TCGA-57-1992-01	**S196P**	Missense_Mutation
STAD	TCGA-BR-6802-01	**V292A**	Missense_Mutation
STAD	TCGA-CG-5723-01	**P158L**	Missense_Mutation
STAD	TCGA-BR-4361-01	**A433Pfs*5**	Frame_Shift_Del
STAD	TCGA-BR-8591-01	**V473Cfs*16**	Frame_Shift_Del
STAD	TCGA-BR-8078-01	**R959Gfs*5**	Frame_Shift_Del
STAD	TCGA-BR-4370-01	**R653W**	Missense_Mutation
STAD	TCGA-BR-8361-01	**W782***	Nonsense_Mutation
STAD	TCGA-BR-8361-01	**V807M**	Missense_Mutation
STAD	TCGA-BR-8678-01	**E822del**	In_Frame_Del
STAD	TCGA-HU-A4GQ-01	**R754H**	Missense_Mutation
STAD	TCGA-VQ-A92D-01	**A733V**	Missense_Mutation
SARC	TCGA-DX-A7EF-01	**R953Q**	Missense_Mutation
UCEC	TCGA-B5-A3FA-01	**R1013C**	Missense_Mutation
UCEC	TCGA-AP-A1DV-01	**R77Q**	Missense_Mutation
UCEC	TCGA-AP-A0LM-01	**F101S**	Missense_Mutation
UCEC	TCGA-AX-A2HG-01	**V473Cfs*16**	Frame_Shift_Del
UCEC	TCGA-B5-A3FC-01	**R510C**	Missense_Mutation
UCEC	TCGA-AP-A1DK-01	**R953***	Nonsense_Mutation
UCEC	TCGA-AJ-A3EK-01	**R653Q**	Missense_Mutation
UCEC	TCGA-AP-A051-01	**W683***	Nonsense_Mutation
UCEC	TCGA-AP-A059-01	**A626V**	Missense_Mutation
UCEC	TCGA-AP-A1DK-01	**S685F**	Missense_Mutation

UCEC	TCGA-AP-A1DK-01	**G982W**	Missense_Mutation
UCEC	TCGA-AP-A1DV-01	**R302H**	Missense_Mutation
UCEC	TCGA-AX-A0J0-01	**V740A**	Missense_Mutation
UCEC	TCGA-AX-A2HD-01	**A884T**	Missense_Mutation
UCEC	TCGA-B5-A5OC-01	**P152T**	Missense_Mutation
UCEC	TCGA-DF-A2KR-01	**K878Q**	Missense_Mutation
UCEC	TCGA-DF-A2KU-01	**L296M**	Missense_Mutation
UCEC	TCGA-EO-A22R-01	**E354D**	Missense_Mutation
UCEC	TCGA-EO-A22S-01	**P496L**	Missense_Mutation
UCEC	TCGA-EO-A22U-01	**V577M**	Missense_Mutation
UCEC	TCGA-EO-A22U-01	**X677_splice**	Splice_Site
UCEC	TCGA-EO-A3B0-01	**R962Q**	Missense_Mutation
UCEC	TCGA-EY-A215-01	**A825T**	Missense_Mutation
UCEC	TCGA-EY-A2OM-01	**A433Gfs*23**	Frame_Shift_Ins
UCEC	TCGA-A5-A1OF-01	**R960W**	Missense_Mutation
UCEC	TCGA-A5-A1OF-01	**A762T**	Missense_Mutation
UVM	TCGA-YZ-A985-01	**G607S**	Missense_Mutation
